# Prospects for Genomic Selection in Cassava Breeding

**DOI:** 10.3835/plantgenome2017.03.0015

**Published:** 2017-09-28

**Authors:** Marnin D. Wolfe, Dunia Pino Del Carpio, Olumide Alabi, Lydia C. Ezenwaka, Ugochukwu N. Ikeogu, Ismail S. Kayondo, Roberto Lozano, Uche G. Okeke, Alfred A. Ozimati, Esuma Williams, Chiedozie Egesi, Robert S. Kawuki, Peter Kulakow, Ismail Y. Rabbi, Jean-Luc Jannink

**Affiliations:** 1 Section on Plant Breeding and Genetics, Cornell Univ., Ithaca, NY; 2 International Inst. for Tropical Agriculture, Ibadan, Oyo, Nigeria; 3 National Root Crops Research Inst., Umudike, Umuahia, Nigeria; 4 National Crops Resources Research Inst., Namulonge, Uganda; 5 USDA-ARS, R.W. Holley Center for Agriculture and Health, Ithaca, NY; 6 International Programs, College of Agriculture and Life Sciences, Cornell Univ., Ithaca, NY

## Abstract

Cassava (*Manihot esculenta* Crantz) is a clonally propagated staple food crop in the tropics. Genomic selection (GS) has been implemented at three breeding institutions in Africa to reduce cycle times. Initial studies provided promising estimates of predictive abilities. Here, we expand on previous analyses by assessing the accuracy of seven prediction models for seven traits in three prediction scenarios: cross-validation within populations, cross-population prediction and cross-generation prediction. We also evaluated the impact of increasing the training population (TP) size by phenotyping progenies selected either at random or with a genetic algorithm. Cross-validation results were mostly consistent across programs, with nonadditive models predicting of 10% better on average. Cross-population accuracy was generally low (mean = 0.18) but prediction of cassava mosaic disease increased up to 57% in one Nigerian population when data from another related population were combined. Accuracy across generations was poorer than within-generation accuracy, as expected, but accuracy for dry matter content and mosaic disease severity should be sufficient for rapid-cycling GS. Selection of a prediction model made some difference across generations, but increasing TP size was more important. With a genetic algorithm, selection of one-third of progeny could achieve an accuracy equivalent to phenotyping all progeny. We are in the early stages of GS for this crop but the results are promising for some traits. General guidelines that are emerging are that TPs need to continue to grow but phenotyping can be done on a cleverly selected subset of individuals, reducing the overall phenotyping burden.

## Core Ideas

- Accuracy is generally similar across breeding populations.
- Data sharing across programs improves predictions in some circumstances.
- Accuracy across generations is sufficient for rapidcycling genomic selection (GS) on several traits.
- Phenotyping small numbers of progeny can have a large impact on prediction accuracy.
- Prospects for GS in cassava are good and improving.

**C**ASSAVA, a root crop with origins in the Amazon basin (Olsen and Schaal, 1999), provides staple food for more than 500 million people worldwide (Howeler et al., 2013). It is widely cultivated in Sub-Saharan Africa, where the storage roots serve as primary source of carbohydrates and can be processed into a wide variety of products such as *fufu*, *lafun*, *gari*, *abacha*, tapioca, and starch (Chukwuemeka, 2007; Bamidele et al., 2015).

Cassava is a diploid (2*n* = 36) and highly heterozygous non-inbred crop that is propagated vegetatively by farmers using stem cuttings, though most genotypes flower and can be used to produce botanical seeds from either self- or cross-pollination. Among the most important traits targeted for improvement are storage root yield, dry matter content (DM), starch content, tolerance to postharvest physiological deterioration, carotenoid content, and resistance to pests or diseases (Esuma et al., 2016).

**Fig. 1:**
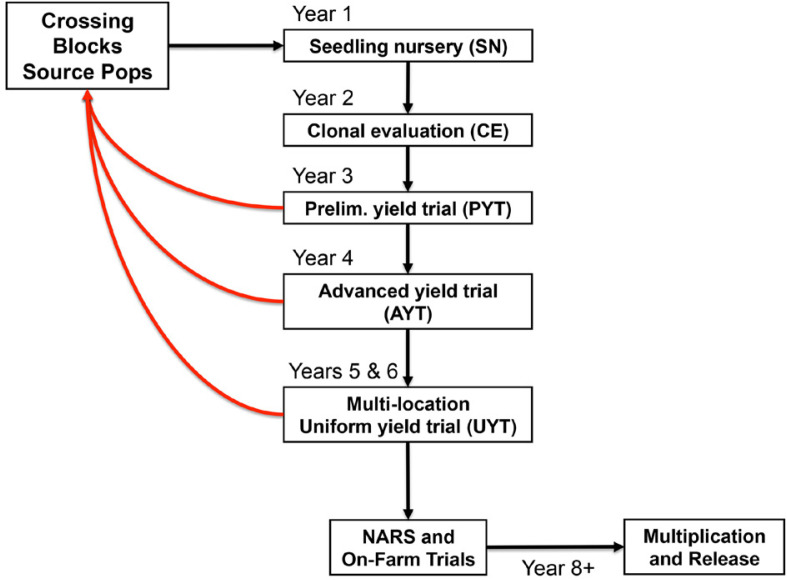
Schematic of a conventional cassava breeding cycle. Arrows between trials indicate the selection of materials for further phenotyping trials. Red arrows indicate the selection of materials as parents for crossing.

Development and implementation of breeding strategies in cassava represent a challenge because of the crop’s heterozygous nature and long breeding cycle. A traditional cassava-breeding program relies on phenotypic characterization of mature plants that have been clonally propagated. Typically, cycles of selection take 3 to 6 yr from seedling germination to multilocation yield trials and additional years are required to evaluate promising genotypes before variety release (Fig. 1).

Marker-assisted selection has been effective in cassava for the selection of promising genotypes for resistance to cassava mosaic disease (CMD) (Okogbenin et al., 2007; Ceballos et al., 2015; Parkes et al., 2015). However, the use of marker-assisted selection is limited primarily to traits with known large-effect loci, which makes this method infeasible for complex traits (Dekkers and Hospital, 2002; Heffner et al., 2009).

With the advent of next-generation sequencing technologies, it is now affordable to profile single nucleotide polymorphism (SNP) markers genome-wide (Barabaschi et al., 2015), which can support the use of GS, a breeding method that uses such markers to predict the breeding values of unevaluated individuals (Meuwissen et al., 2001). Genomic selection can optimize and accelerate pipelines for population improvement and variety development and release (Heffner et al., 2009) with a reduction in breeding time resulting from selection of parental genotypes with superior breeding values at the seedling stage based on genotypes alone.

Many genomic prediction models are available, differing from each other primarily with respect to the genetic architecture that they assume. For example, genomic best linear unbiased prediction (GBLUP) assumes an infinitesimal genetic architecture (nearly equal and small contributions by all genomic regions to the phenotypes). In contrast, models like BayesB alter that assumption, putting emphasis on major-effect loci and variable selection (Gianola et al., 2009; Legarra et al., 2011; Habier et al., 2011). Evaluation of different GS models with nonsimulated data indicates that prediction accuracy varies across species and traits (Heslot et al., 2012; Resende et al., 2012; Gouy et al., 2013; Charmet et al., 2014; Rutkoski et al., 2014; Cros et al., 2015).

Previous studies in cassava have estimated genetic parameters and evaluated prediction accuracy by applying the GBLUP model with small training sets and low-density markers (Oliveira et al., 2012, 2014). Historical phenotypic data from the International Institute of Tropical Agriculture (IITA), combined with markers obtained from genotyping-by-sequencing (GBS), showed promising results for cassava breeding with GS (Ly et al., 2013). In that study, the predictive ability (accuracy) was measured as the correlation between predictive values and the phenotypic value ranged from 0.15 to 0.47 across traits (Ly et al., 2013).

There are ongoing efforts within the Next Generation Cassava Breeding project (www.nextgencassava.org, accessed 14 Aug. 2017) to increase the rate of genetic improvement in cassava and unlock the full potential of cassava production. The project is currently in the early stages of implementing GS at three African research institutes: the National Crops Resources Research Institute (NaCRRI) in Uganda, the National Root Crops Research Institute (NRCRI) in Nigeria, and the IITA, also in Nigeria.

In the present study, we evaluated the potential of GS as a breeding tool to increase the rates of genetic gain in datasets associated with all three Next Generation Cassava Breeding breeding programs. We assessed predictive ability by cross-validation within TP datasets for seven traits: DM, fresh root weight (RTWT), root number (RTNO), shoot weight (SHTWT), harvest index (HI), severity of CMD (MCMDS), and plant vigor. We compared the performance of seven GS models for these traits in each of the breeding programs.

One important topic in GS concerns the feasibility of prediction across generations and across TPs from different breeding populations or programs. To increase the rate of gain achievable by GS, prediction models will need to accurately rank unevaluated progenies rather than genotypes contemporary with the TP. It is well known that recombination and divergence relative to the TP associated with recurrent selection reduces the accuracy of cross-generation prediction, making this kind of prediction a major challenge for GS (Jannink, 2010; Lorenz et al., 2011). Accuracies in these scenarios have not been previously estimated in cassava. Therefore, we tested the accuracy of cross-generation prediction with the IITA TP and two successive cycles of progeny that have been phenotyped. Similarly, given that the previous results indicated only a small level of genetic differentiation among clones from different populations (Wolfe et al., 2016b), we tested whether combining information from different populations could increase prediction accuracy in the smaller populations.

Finally, in a typical scenario, a GS program will phenotype all selected materials and a subset of the unselected material to update the training model. We further investigated the impact of phenotyping different sized subsets of materials for the TP update. We compared random subset selections to selections based on a TP optimization algorithm (Akdemir et al., 2015).

This study is a starting point for successful application of GS in African cassava. Similar to other studies, factors such as trait heritability, the prediction model, and TP composition play important roles in determining the prediction accuracy and the rate the of genetic progress. For example, traits with higher heritability like DM are considered to be more likely to respond to selection and lead to larger genetic gain over cycles of selection (Kawano et al., 1998; Ceballos et al., 2015). Our results will serve to guide implementation strategies for GS in cassava breeding programs.

## Materials & Methods

### Germplasm

In this study, we analyzed data from the GS programs at three African cassava breeding institutions: NaCRRI, NRCRI, and IITA. Germplasm from NaCRRI included 411 clones descended from crosses among accessions from East Africa, West Africa, and South America. The collection from NRCRI was made up of 899 clones, 211 of them in common with the IITA breeding germplasm. The remaining 688 clones were materials derived either in part or directly from the International Center for Tropical Agriculture in Cali, Columbia. Wolfe et al. (2016b) shows details of the origins and pedigrees of the NaCRRI and NRCRI clones used in this study.

The primary IITA germplasm we analyzed is also known as the Genetic Gain (GG) collection, which comprises 709 elite and historically important breeding clones and a few landraces that have been collected starting in the 1970s. These materials have also been previously described in Okechukwu and Dixon (2008), Ly et al. (2013), and Wolfe et al. (2016b).

**Fig. 2:**
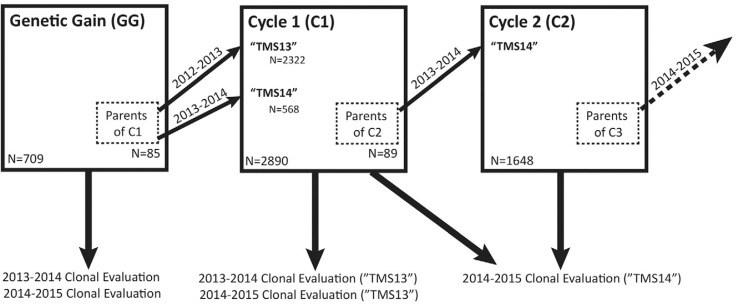
Schematic of International Institute of Tropical Agriculture (IITA) genomic selection, 2012–2015. Three generations of the IITA genomic selection program are illustrated here. From the genetic gain (GG) population, 85 parents were selected and crosses over 2 yr (‘TMS13F’ in 2012–2013 and ‘TMS14F’ in 2013–2014) gave rise to 2890 Cycle 1 (C1) progeny. Predictions based on data from the GG were used to select 89 parents from among C1 in 2013, giving rise to 1648 Cycle 2 (C2) progeny in 2014. The GG were clonally evaluated in 2013–2014 and 2014–2015. The ‘TMS13’ C1 progeny were evaluated in 2013–2014 and 2014–2015. The ‘TMS14’ C1 progeny were evaluated with the C2 progeny in 2014–2015.

In addition, two generations of GS progeny were analyzed (Fig. 2). The parents of each set of progeny were chosen on the basis of their GEBVs as described previously (Wolfe et al., 2016b). The first, GS cycle 1 (C1) comprised 2890 clones from 166 full-sib families with 85 parents from the GG collection. Because of inconsistency in the timing and amount of flowering and seed set among clones, successful crossing is a challenge in cassava. To obtain the full set of desired matings among parents of C1, crossing blocks were planted in two successive years (2013 and 2014). In 2013, 79 parents produced 2322 seedlings (135 full-sib families). In 2014, 17 parents, 11 of which were reused from the previous year and another six of which were new parents from the GG collection, gave rise to an additional 568 seedlings (31 new full-sib families). The C1 families had a mean size of 17.4 siblings (median: 15, range: 2–78).

Finally, in 2014, a crossing block was planted with 89 selected C1 parents, which generated 1648 GS Cycle 2 (C2) seedlings in 242 full-sib families. The Cycle 2 families had a mean size of 6.8 individuals (median: 6, range: 1–20).

### Phenotyped Traits

Seven traits were analyzed in this study. Plant vigor was recorded as 3 = low, 5 = medium, and 7 = high 1 mo after planting at IITA and NRCRI and 3 mo after planting at NaCRRI. We used the across-season average MCMDS for our analyses; this was the mean of measurements taken at 1, 3, and 6 mo after planting, on a scale of 1 (no symptoms) to 5 (severe symptoms). Dry matter content was expressed as a percentage of dry root weight relative to fresh root weight (RTWT). At IITA, DM was measured by drying 100 g of fresh roots in an oven, whereas at NRCRI and NaCRRI, the specific gravity method (Kawano et al., 1987) was used. Root weight and SHTWT were expressed in kilograms per plot, whereas HI was the proportion of total biomass per plot (i.e., RTWT). Root number was the number of fresh roots harvested per plot. For all analyses below, RTNO, RTWT, and SHTWT were natural-log transformed to obtain normally distributed residuals.

The phenotyping trials analyzed in this study have been described in part in previous publications (Wolfe et al., 2016a; 2016b). However, complete details on the phenotyping trial design particular to this study are provided in Supplemental File S1. All phenotyping trials were conducted between 2013 and 2015. Clones from NaCRRI were evaluated in three locations with different agro-ecological conditions in Uganda: Namulonge, Kasese, and Ngetta. Clones from NRCRI were tested in three locations in Nigeria: Kano, Otobi, and Umudike. Meanwhile, IITA clones were evaluated in four locations within Nigeria: Ibadan, Ikenne, Ubiaja, and Mokwa.

### Two-Stage Genomic Analyses

Except where noted otherwise, a two-step approach was used to evaluate genomic predictions in this study. This approach was used to correct for heterogeneity in the experimental designs and increase computational efficiency. The first stage involved accounting for trial design-related variables with a linear mixed model.

For NaCRRI we fitted the model shown in Eq. [1]:

$$y=X{\beta +Z}_{\text{clone}}c+Z_{\text{range(loc.year)}}r+Z_{\text{block(range)}}b+\text{ε},$$ 1

where **β** included a fixed effect for the population mean, the location–year combination, and for plot-basis traits (RTWT, RTNO, and SHTWT); the number of plants harvested per plot was included as a covariate; the vector *c* and the corresponding incidence matrix **Z_clone_** represented a random effect for the clone where *c* ~ N(0,**I**${\text{σ}}_{c}^{\text{2}}$); **I** represented the identity matrix; and the range variable was nested in location–year–replication and was represented by the incidence matrix **Z_range(loc.year)_** and the random effects vector *r* ~ N(0,**I**${\text{σ}}_{r}^{\text{2}}$). Ranges were equivalent to the row or column along which plots were arrayed. Blocks were also modeled, with a block being a subset of a range. Block effects were nested in ranges and were incorporated as random variables with the incidence matrix **Z_block(range)_** effects vector *b* ~ N(0,**I**${\text{σ}}_{b}^{\text{2}}$). Finally, the residuals ε were random, with ε ~ N(0,**I**${\text{σ}}_{\text{ε}}^{\text{2}}$).

The model for NRCRI was:

$$y={\text{Xβ + Z}}_{\text{clone}}c+{\text{Z}}_{\text{set(loc.year)}}s+{\text{Z}}_{\text{rep(set)}}r+{\text{Z}}_{\text{block(rep)}}b+\text{ε},$$ 2

where **Z_set_** was the incidence matrix corresponding to the random effect for the planting group (see above), which was nested in location–year, with *s* ~ N(0,**I**${\text{σ}}_{s}^{\text{2}}$). Replication effects were nested in sets and treated as random with the incidence matrix **Z_rep(set)_** and the effects vector *r* ~ N(0,**I**${\text{σ}}_{r}^{\text{2}}$). Blocks were nested in replications, treated as random, and represented by the design matrix **Z_block(rep)_** and the effects vector *b* ~ N(0,**I**${\text{σ}}_{b}^{\text{2}}$). The fixed effects for NRCRI included were the same as those for NaCRRI, with the addition of a term for trial (i.e., TP1 and TP2; see above).

For IITA, data from all trials described above were fitted together using the model in Eq. [3]:

$$y={X\beta +\text{Z}}_{\text{clone}}c+{\text{Z}}_{\text{range(loc.year)}}r+\text{ε.}$$ 3

The range effect was fitted as random. The fixed effects were the same as those described for NaCRRI, except the proportion of harvested plants (out of the total originally planted) was used instead of the number harvested as a cofactor. This was done to correct for differences in plot sizes.

For the clone effect, the best linear unbiased prediction (BLUP) (***ĉ***), which represents an estimate of the total genetic value (estimated genetic value, EGV) for each individual, was extracted. The EGVs were de-regressed by dividing by their reliability $\text{(1–}\frac{\text{PEV}}{{\text{σ}}_{c}^{\text{2}}}\text{)}$, where PEV is the prediction error variance of the BLUP. This was done to avoid applying shrinkage to the same data twice (once in the first step and again in the genomic prediction step). The mixed models above were solved with the *lmer* function of *lme4* package (Bates et al., 2014) in R (https://cran.r-project.org, accessed 30 Aug. 2017).

We used de-regressed the EGVs as the response variables and weighted error variances in downstream genomic evaluations. Error variances were weighted according to Garrick et al. (2009) via Eq. [4]:

$$\frac{1}{\sqrt{\frac{1-H^{2}}{0.1+\frac{1-r^{2}}{r^{2}}H^{2}}},}$$ 4

where *H*^2^ is the proportion of the total variance explained by the clonal variance component, ${\text{σ}}_{c}^{2}$. Weighting error variances during the genomic prediction step was done to preserve information from the first step about differences between clones in the reliability of the de-regressed BLUPs being used to represent their genetic value. These differences occur mostly because of imbalances in the number of observations among clones. This information would otherwise be ignored when making genomic predictions with a two-step procedure.

### Genotyping Data

The cassava collections described above were genotyped with GBS (Elshire et al., 2011) with the *ApeKI* restriction enzyme recommended by Hamblin and Rabbi (2014). Single nucleotide polymorphisms were called with the TASSEL 5.0 GBS pipeline version 2 (Glaubitz et al., 2014) and aligned to the cassava reference genome, version 6.1 (http://phytozome.jgi.doe.gov, accessed 14 Aug. 2017; International Cassava Genetic Map Consortium, 2015). Genotype calls were only allowed when a minimum of two reads was present; otherwise, the genotype was imputed (see below). Furthermore, the GBS data were filtered so that clones with >80% missing and markers with >60% missing genotype calls were removed. Markers with extreme deviation from Hardy–Weinberg equilibrium (*Χ*^2^ > 20) were also removed. Only biallelic SNP markers were considered for further analyses. We used a combination of custom scripts and common variant call file (Danecek et al., 2011) manipulation tools to accomplish this pipeline. Finally, imputation was conducted with Beagle version 4.0 (Browning & Browning, 2009). A total of 155,871 markers were obtained following these procedures. For genomic prediction in a given population or dataset, we further filtered out SNPs with a minor allele frequency less than 0.01.

### Assessment of Prediction Accuracy via Cross-Validation

To obtain unbiased estimates of prediction accuracy, we used a *k*-fold cross-validation scheme (Kohavi, 1995). In brief, each breeding program dataset [NRCRI collection (NR), NaCRRI collection (UG), and GG] was split randomly into *k* = fivefold mutually exclusive training and validation sets. The training set composed of four out of five of the subsets was used to estimate marker effects for predictions. The estimated marker effects were used to predict the breeding value of the validation set individuals. The process of subset assignment and genomic prediction was repeated 25 times for each model. For each repeat, predictions were accumulated from each individual when it was in the validation subset. Prediction accuracy was then calculated as the Pearson correlation between the EGV (not de-regressed) and the accumulated predicted values for that repeat.

### Genomic Prediction Methods

In this study, we compared the accuracy of genomic prediction via seven methods that are briefly described below. These methods differ in their assumptions about genetic architecture and whether the prediction being made represents a genome estimated breeding value (GEBV that included additive effects or a genome estimated total genetic value, which includes additive and nonadditive effects. Prediction models were compared by examining several prediction scenarios (described in detail below), including 25 replications of fivefold cross-validation, crossgeneration, and cross-population prediction.

#### Genomic BLUP

Prediction with GBLUP involves fitting a linear mixed model of the following form: ***y*** = **Xβ** + **Z*g*** + **ε**. Here, ***y*** is a vector of the phenotype and **β** is a vector of fixed, nongenetic effects with the design matrix **X.** The vector ***g*** is a random effect, the BLUP, which represents the GEBV for each individual. **Z** is a design matrix indicating observations of genotype identities, and ***ε*** is a vector of residuals. The GEBV is obtained by assuming ***g*** ~ N(0,**K**${\text{σ}}_{g}^{\text{2}}$, where ${\text{σ}}_{g}^{2}$ is the additive genetic variance and **K** is the square, symmetric genomic realized relationship matrix based on SNP markers. The genomic relationship matrix was constructed with the function *A.mat* in the R package rrBLUP (Endelman, 2011) and follows the formula of VanRaden (2008), Method 2. Predictions using GBLUP were made with the function *emmreml* in the R package EMMREML (Akdemir and Okeke, 2015).

#### Reproducing Kernel Hilbert Spaces

We made predictions with reproducing kernel Hilbert spaces (RKHS). The genomic relationship matrix used in the GBLUP model described above can be considered as a parametric (additive genetic) kernel function and exists as a special case of RKHS (Gianola and van Kaam, 2008; Morota and Gianola, 2014). For RKHS predictions, we used a mixed model of the same form as for GBLUP above. Unlike the case of GBLUP, we used a Gaussian kernel function:

$$K_{ij}\;=\;\exp(-(d_{\text{ij}}\text{θ})),$$ 5

where *K*_ij_ was the measured relationship between two individuals, *d*_ij_ was their Euclidean genetic distance based on marker dosages, and θ was a tuning (sometimes called a “bandwidth”) parameter that determines the rate of decay of correlations among individuals. Because this is a nonlinear function, the kernels we used for RKHS could capture nonadditive as well as additive genetic variation. Thus the BLUPs from RKHS models represent genome estimated total genetic values rather than GEBVs.

Because the optimal θ must be determined, a range of values was tested in two ways. First, we did cross-validation with the following θ values and selected the one with the best accuracy: 0.0000005, 0.000005, 0.00005, 0.0001, 0.0005, 0.001, 0.004, 0.006, 0.008, 0.01, 0.02, 0.04, 0.06, 0.08, and 0.1 (single-kernel RKHS). Second, we used the *emmremlMultiKernel* function in the EMMREML package (Akdemir and Okeke, 2015) to fit a multikernel model with six covariance matrices, with the following bandwidth parameters and allowed restricted maximum likelihood to find optimal weights for each: 0.0000005, 0.00005, 0.0005, 0.005, 0.01, and 0.05 (multikernel RKHS).

#### Bayesian Marker Regressions

We tested four well-established Bayesian prediction models: BayesCpi (Habier et al., 2011), the Bayesian LASSO (BL; Park and Casella, 2008), BayesA, and BayesB (Meuwissen et al., 2001). In ridge-regression (equivalent to GBLUP), marker effects were all shrunk by the same amount, because we assume they are all drawn from a normal distribution with the same variance. Further, all markers have a nonzero effect and most have small effects, essentially assuming that the genetic architecture of the trait is infinitesimal. In contrast, the Bayesian models we tested allow for alternative genetic architectures by inducing differential shrinkage of marker effects. For BayesA and Bayesian LASSO, all markers have a nonzero effect but the marker variances are drawn from scaled-*t* and double-exponential distributions respectively, which are both distributions with thicker tails and greater density at zero. Both BayesB and BayesCpi are variable selection models because the marker variances come from a two-component mixture of a point mass at zero and either a scaled-*t* distribution (BayesB) or a normal distribution (BayesCpi). Fitting BayesB and BayesCpi begins by estimating a parameter *pi*, representing the proportion of markers with a nonzero effect. We performed Bayesian predictions with the R package BGLR (Pérez and De Los Campos, 2014). Following Heslot et al. (2012) and others, we ran BGLR for 10,000 iterations, discarded the first 1000 iterations as burn-in, and thinned the remainder to every fifth sample. Marker dosages were mean-centered on the combination of training and test sets before analysis. Convergence was confirmed visually in initial test runs using the CODA package in R (Plummer et al., 2006).

#### Random Forest

Random Forest (RF) is a machine learning method used widely in regression and classification (Breiman, 2001; Strobl et al., 2009). The use of RF regression with marker data has been shown to capture epistatic effects and has been successfully used for prediction of genome estimated total genetic value (Breiman, 2001; Motsinger-Reif et al., 2008; Michaelson et al., 2010; Heslot et al., 2012; Charmet et al., 2014; Sarkar et al., 2015; Spindel et al., 2015). In prediction, a random forest is a collection of *r* regression trees grown on a subset of the original dataset that is bootstrapped over observations and randomly sampled over predictors. Averaging the prediction over trees for validation observations then aggregates the information. We used RF with the parameter with *ntree* set to 500 and the number of variables sampled at each split (*mtry*) equal to 300. We implemented RF with the randomForest package in R (Liaw and Wiener, 2002). As in the Bayesian regressions, marker dosages were mean-centered before RF analysis.

### Comparison of Models Based on the Similarity of Rankings

To test for GS model similarities among breeding programs, we clustered the GEBV output on a breeding program basis. Genomic estimated breeding values from each model were scaled and centered on a column basis with the *scale* function in R and were then used to construct a matrix of Euclidean distances between models. Distance matrices were used as an input for hierarchical clustering using the Ward criterion implemented in the *hclust* R function (Heslot et al., 2012).

### Cross-Generation Genomic Predictions

Because nearly all of the IITA germplasm from C1 and C2 had been clonally evaluated, we were able to test the prospects for predicting unevaluated progeny. We predicted all traits via all methods in four scenarios: GG predicting C1, GG predicting C2, C1 predicting C2, and GG + C1 predicting C2. Unlike the other predictions presented in this study, cross-generation predictions were done in a single step (raw phenotype and genomic data were fitted simultaneously). The exception was for RF, where correction for location and blocking factors is not supported. For RF prediction, we used the same de-regressed EGVs as for cross-validation. The software and parameters used were the same as already described. The design model was the same as that described for IITA above.

### Training Population Update

We evaluated the impact on cross-generation prediction accuracy of phenotyping different size subsets of the unselected C1 (materials selected for crossing in each cycle were phenotyped, but unselected materials were not phenotyped in all cases). We selected subsets of C1 using two methods: randomly and with a genetic algorithm implemented in the R package STPGA (Akdemir et al., 2015).

The STPGA package uses an approximation of the mean PEV expected for a given set of training individuals in combination with a given set of test genotypes as a criterion (which does not require phenotype data) for selecting the “optimal” training set. The genetic algorithm implemented by STPGA is used to rapidly find the training set that minimized the selection criterion (the mean PEV of the test set; Akdemir et al., 2015). To speed up computation, STPGA uses principal components rather than raw SNP markers as genetic predictors.

**Fig. 3:**
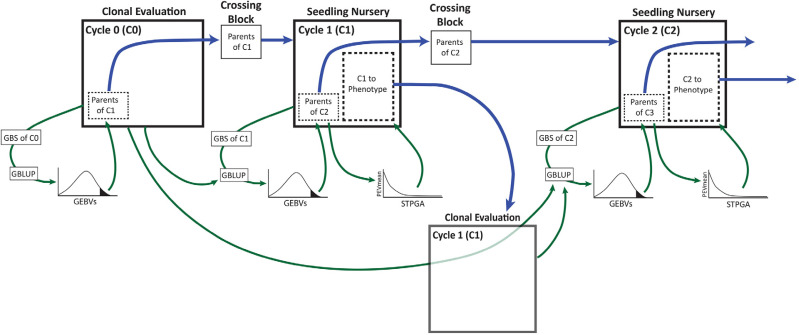
Schematic of genomic selection with training population optimization carried out by STPGA. The selection was initially made among available genotyped candidates on the basis of genomic predictions with available phenotype data. Selected parents are grown and mated in a crossing block. The resulting Cycle 1 (C1) seeds are subsequently collected and grown in a nursery. Cycle 1 seedlings were genotyped by genotyping-by-sequencing (GBS) and selections were made on the basis of genomic prediction alone. Selected parents of Cycle 2 (C2) were cloned into a crossing nursery. STPGA was used to select the optimal additional C1 seedlings to plant in a clonal evaluation trial. Because C2 seedlings do not yet exist, STPGA was instead used to select the optimal C1 seedlings to predict the selected parents of C2. Phenotypes from the C1 clonal evaluation were added to the existing genomic prediction training dataset. The updated training model will be used to predict breeding values of theC2 seedlings when the GBS data become available and the selections of the parents of Cycle 3 (C3) is made. Subsequent cycles will proceed following this procedure.

Parents selected for further recombination were cloned into a crossing block. This was the point at which additional unselected seedlings must be chosen for phenotyping to incorporate their data in predictions of the eventual progeny that are produced. Since the next generation of progeny had not yet been produced, we targeted STPGA on the parents of C2. Figure 3 provides a schematic of GS with the TP update and optimization with STPGA. We constructed a genomic relationship matrix with only C1 (including the parents of C2). We did a principal component analysis on the kinship matrix and took the first 100 principal components as genomic predictors. We ran 1000 iterations of the genetic algorithm 10 times at each sample size. Sample sizes ranged from 200 to 2400 at increments of 400 (Supplemental Table S1). Predictions at each sample size were then made with each of 10 random and 10 optimized training sets using GBLUP in two scenarios: either just the sample of C1 was used to train the model or the sample of C1 plus all of GG were used.

### Cross-Population Genomic Predictions

We predicted all traits using all methods in three scenarios: GG + NR predicting UG, GG + UG predicting NR, and NR + UG predicting GG (Supplemental Table S2A). Cross-population predictions were made with the prediction models described above and followed the two-step approach as described above.

We selected optimized subsets of the combined datasets with a genetic algorithm implemented in the R package STPGA (Akdemir et al., 2015). Random subsets of the same size as the optimized subsets (300, 600, 900, and 1200) were selected for comparisons between predictive accuracies. Predictions at each sample size were then made for 10 random and 10 optimized training sets with GBLUP.

## Results

After quality control and keeping only markers with >1% minor allele frequency, the datasets had between 70,010 and 78,212 SNP markers (Table 1). Principal component analysis of the genomic relationship matrix indicated some genetic differentiation between Nigerian populations (GG and NR) and the Ugandan TP (UG; Supplemental Fig. S1a). In contrast, there was little differentiation between the NRCRI and IITA GG datasets, even when we compared only the nonoverlapping clones. We also calculated the *F*_ST_ between populations as implemented in *vcftools* (Danecek et al., 2011). In agreement with results from the principal component analysis, the F_ST_ between GG and NR was only 0.008, but was 0.019 and 0.021 between the Ugandan and the Nigerian populations, GG and NR, respectively. There was a similar amount of genetic differentiation between the IITA C2 progeny and its grandparental GG population (F_ST_ = 0.02), as there was between GG and UG (Table 1, Supplemental Fig. S1b).

**Table 1:** Summary and comparison of phenotype and genotype datasets analyzed in this study.

Broad-sense heritability						
Trait	IITA¶				NRCRI	NaCRRI
	All IITA	GG	C1	C2		
VIGOR	0.25	0.25	0.31	0.19	0.06	0.15
MCMDS	0.69	0.60	0.86	0.25	0.44	0.62
DM	0.49	0.59	0.62	0.51	0.01	0.14
HI	0.57	0.36	0.62	0.55	0.12	0.36
RTWT	0.31	0.10	0.36	0.00	0.10	0.27
RTNO	0.24	0.09	0.26	0.00	0.06	0.22
SHTWT	0.22	0.14	0.21	0.00	0.13	0.25
No. Clones	5247	709	2890	1648	899	411
Raw data points	8501	2924	3875	1702	2391	7662

**Genetic diversity statistics**						

Mean Inbreeding Coefficient†		0.933	0.965	0.949	0.946	0.954
Std Dev. Kinship Coefficient‡		0.080	0.089	0.092	0.080	0.118
MAF > 1%		76137	73096	70010	78212	75923
Median (MAF)		0.009	0.0067	0.0047	0.01	0.01
Mean Heterozygosity§		0.16	0.15	0.17	0.15	0.15
Max. Heterozygosity		0.29	0.27	0.28	0.26	0.24
Min. Heterozygosity		0.07	0.07	0.10	0.07	0.08
Mean (MAF)		0.056	0.054	0.056	0.055	0.054

**Mean F_ST_ between datasets**						
**Populations compared**	**F_ST_**	**Populations compared**		**F_ST_**		
		
GG vs. NR	0.008	GG vs. C1		0.010		
GG vs. UG	0.019	GG vs. C2		0.020		
NR vs. UG	0.021	C1 vs. C2		0.014		

† Mean of the diagonal of the genomic relationship matrix.

‡ Off-diagonal of the genomic relationship matrix.

§ Heterozygosity per individual per dataset.

¶ IITA, International Institute of Tropical Agriculture; GG, IITA Genetic Gain germplasm collection; C1, IITA Cycle 1; C2, IITA Cycle 2; NR, National Root Crops Research Institute (NRCRI); UG, National Crops Resources Research Institute (NaCRRI); DM, dry matter content; HI, harvest index; RTWT, root weight; RTNO, root number; SHTWT, shoot weight; MCMDS, mean cassava mosaic disease severity; VIGOR, early plant vigor;MAF, minor allele frequency.

The mean inbreeding coefficient (F), as measured by the mean of the diagonal of the genomic relationship matrix, was similar for all populations, ranging from 0.933 in GG to 0.965 in C1. The mean rate of heterozygous loci was also similar between populations, ranging from 0.15 to 0.17. There was no notable decrease in heterozygosity or increase in the inbreeding coefficient from GG to C1 or from C1 to C2 (Table 1; Supplemental Fig. S2).

In general, broad-sense heritability was highest in the C1 (mean = 0.46 across traits), lowest for NRCRI (mean = 0.13), and similar for the IITA GG, and NaCRRI TPs. Averaging across populations, broad-sense heritability was highest for MCMDS (0.57), followed by HI (0.43) and DM (0.39). However, broad-sense heritability was generally low for yield components (Table 1).

### Prediction Within Breeding Populations

We tested seven genomic prediction models that differed in their extent and the kind of shrinkage, which is relevant in modelling different genetic architectures, and in their ability to capture nonadditive effects (Supplemental Fig. S3 to Supplemental Fig. S5).

Overall, breeding populations exhibited differences in the cross-validated prediction accuracies between methods and across traits (Table 2 and Supplemental Fig. S3 to Supplemental Fig. S5). For NRCRI (*n* = 899), the mean predictive accuracy values across methods ranged between -0.02 for plant vigor and 0.27 for HI. For NaCRRI (*n* = 411), the mean predictive accuracy values ranged between 0.23 for SHTWT and 0.46 for HI. Meanwhile, the predictive accuracy values for GG (*n* = 709) ranged between 0.22 for plant vigor and 0.66 for DM.

**Table 2:** Summary of cross-validated predictive accuracies by prediction model, trait, and breeding program. The highest predictive accuracy across methods within a trait and within a breeding program is indicated in bold.

Trait	Program	BayesA	BayesB	BayesC	BL‡	GBLUP	Multi- kernel-RKHS	Random Forest	Mean
	NRCRI	0.12	0.12	0.11	0.12	0.10	**0.18**	0.15	0.13
DM	NaCRRI	0.29	0.29	0.30	0.29	0.30	0.33	**0.34**	0.31
	GG	0.67	0.67	0.67	**0.68†**	0.67	0.67	0.63	0.66
	NRCRI	0.27	0.26	0.27	0.24	0.27	0.30	**0.31**	0.27

HI	NaCRRI	0.46	0.45	0.45	0.45	0.45	**0.48†**	0.47	0.46
	GG	0.37	0.39	0.39	0.40	0.39	**0.41**	0.39	0.39
	NRCRI	0.23	0.22	0.23	0.24	0.22	0.32	**0.34**	0.26

RTWT	NaCRRI	0.31	0.30	0.30	0.29	0.31	**0.37†**	0.35	0.31
	GG	0.31	0.31	0.33	0.33	0.32	0.33	**0.34**	0.33
	NRCRI	0.19	0.18	0.18	0.19	0.18	**0.21**	0.20	0.19

RTNO	NaCRRI	0.35	0.34	0.34	0.30	0.35	**0.39†**	0.36	0.34
	GG	0.33	0.33	0.34	0.35	0.35	0.34	**0.35**	0.34
	NRCRI	0.18	0.19	0.19	0.19	0.17	**0.25**	0.24	0.20

SHTWT	NaCRRI	0.21	0.22	0.22	0.18	0.24	**0.26**	0.25	0.23
	GG	0.31	0.32	0.32	**0.33†**	0.32	**0.33†**	0.29	0.31
	NRCRI	0.23	0.22	0.20	0.21	0.19	0.24	**0.29**	0.23

MCMDS	NaCRRI	0.50	**0.50**	0.42	0.41	0.40	0.45	0.48	0.45
	GG	0.58	**0.60†**	0.57	0.56	0.56	0.57	**0.60†**	0.57
	NRCRI	–0.03	–**0.02**	–**0.02**	–0.03	–**0.02**	–0.03	–0.03	–0.02

VIGOR	NaCRRI	0.35	0.34	0.34	0.34	0.35	**0.38†**	**0.38†**	0.34
	GG	0.23	0.23	0.24	**0.24**	0.23	0.22	0.18	0.22

Mean		0.31	0.31	0.30	0.30	0.30	0.33	0.33	

† The highest predictive accuracy within a trait across breeding programs.

‡ BL, Bayesian Lasso; GBLUP, genomic best linear unbiased prediction; RKHS, reproducing kernel Hilbert spaces; GG, International Institute of Tropical Agriculture Genetic Gain germplasm collection; NRCRI, National Root Crops Research Institute; NaCRRI, National Crops Resources Research Institute; DM, dry matter content; HI, harvest index; RTWT, root weight; RTNO, root number; SHTWT, shoot weight; MCMDS, mean cassava mosaic disease severity; VIGOR, early plant vigor.

In the NRCRI population, RKHS and RF, which capture nonadditive effects, had the highest predictive accuracy values for all traits except plant vigor. The trait with the highest predictive accuracy was RTWT (RF (0.34)) and the lowest predictive accuracy was found for vigor (multi-kernel RKHS (–0.03)).

In the NaCRRI population, the multikernel RKHS model showed the highest predictive accuracies for all traits except for CMD, for which BayesB showed the highest value (*r* = 0.50). In this population, CMD had the overall highest predictive accuracy across traits, whereas SHTWT exhibited the lowest predictive accuracy (Bayesian LASSO, *r* = 0.18).

In the IITA GG population, Bayesian approaches performed better for vigor, CMD, SHTWT, and DM, but the RKHS method showed higher predictive accuracies for HI and for yield related traits such as RTWT and RTNO. Meanwhile, RF gave better predictive accuracy when it was used to estimate GEBVs.

Some trait–dataset combinations exhibited better predictive accuracies than others. For example, NaCRRI population had better predictive accuracies for yield components like HI, RTWT and RTNO but the highest predictive values for CMD and DM were obtained in the GG population.

**Fig. 4:**
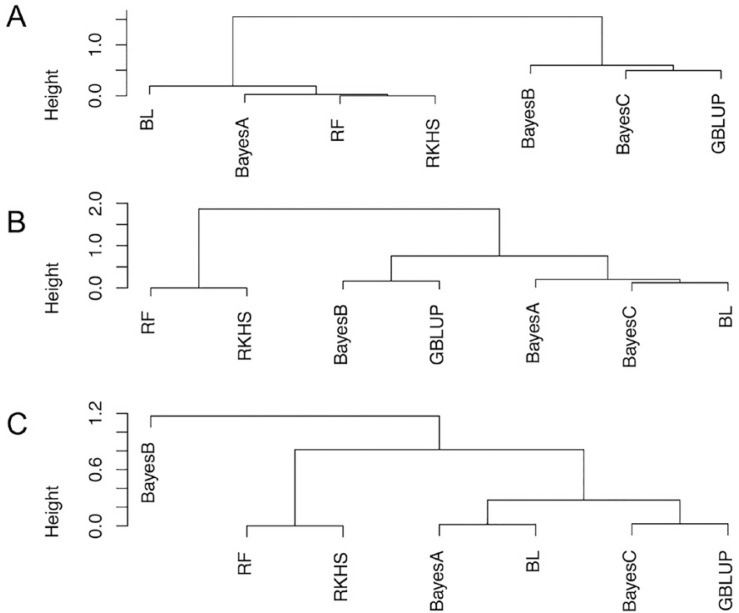
Hierarchical clustering of genomic prediction models based on cross-validated genomic estimated breeding values (GEBVs). Height on the *y*-axis refers to the value of the dissimilarity criterion. (A) Clustering of prediction models in the National Root Crops Research Institute (NRCRI) population. (B) Clustering of prediction models in the National Crops Resources Research Institute (NaCRRI) population. (C) Clustering of prediction models in the Genetic Gain (GG) population. GBLUP, genomic best linear unbiased predictor; BL, Bayesian Lasso; RF, random forest; RKHS, reproducing kernel Hilbert spaces multikernel model.

Similar to Heslot et al. (2012), we compared the cross-validated GEBVs following a clustering approach. The results in Supplemental Fig. S6 show the hierarchical cluster trees from the combined results of the three breeding populations. Differences in the clustering of methods are observed across datasets (Fig. 4). In the NRCRI data, we found two groups of clustering GS methods, with BayesB, BayesC, and GBLUP in one group and the rest on the other group. In the NaCRRI and IITA populations, nonparametric methods such as RKHS and RF clustered together, BayesA clustered with Bayesian LASSO, and GBLUP clustered with BayesC or BayesB.

### Cross-Population Prediction

Previous studies have reported close relatedness between the clones in the next-generation TPs (Wolfe et al., 2016b). One important question within this project is whether or not datasets from different breeding programs can be combined in a training set to increase predictive accuracy. The application of any prediction model with the combined dataset would then benefit from an increase in the TP size with the prospect of using such models in other cassava breeding programs in Africa. With that in mind, we used combined datasets of GG + NR, GG + UG, and UG + NR to predict the population that was not included in the training set (UG, NR, and GG, respectively).

When we predicted the traits in the UG dataset with the combined GG + NR full set, Bayesian models gave better predictive accuracies for MCMDS, RTNO, and DM. Random Forest gave better predictive accuracies for HI and RKHS was best for RTWT and SHTWT (Supplemental Table S2a).

The average predictive accuracy with the combined GG + NR full set as the training set with the GBLUP model was consistently lower for all the traits than the average GBLUP cross-validation results (Supplemental Table S2a). Furthermore, the subsets selected by STPGA to predict the NaCRRI (UG) validation set gave, for all traits and all subset sizes, lower predictive accuracies than the GBLUP cross-validation model (Table 3; Supplemental Fig. S7; Supplemental Table S2b).

**Table 3:** Summary of mean genomic best linear unbiased prediction (GBLUP) cross-validated predictive accuracies across populations. Four subset selection methods (random vs. STPGA) and the full set were considered. The highest predictive accuracy across subsets and the full set is indicated in bold.

Train	Test	Trait	300		600		900		1200		Full	CVGBLUP†
			STPGA	Random	STPGA	Random	STPGA	Random	STPGA	Random		
NR + GG	UG	VIGOR	0.199	0.083	0.182	0.102	**0.221**	0.152	0.200	0.174	0.193	0.353
NR + GG	UG	MCMDS	**0.293**	0.224	0.284	0.264	0.262	0.279	0.284	0.291	0.285	0.404
NR + GG	UG	DM	0.272	0.209	0.282	0.227	0.258	0.254	0.252	0.272	**0.284**	0.296
NR + GG	UG	HI	**0.294**	0.176	0.278	0.230	0.266	0.215	0.228	0.214	0.206	0.454
NR + GG	UG	RTWT	0.155	0.072	0.165	0.124	0.181	0.156	0.179	0.174	**0.193**	0.314
NR + GG	UG	RTNO	0.149	0.068	0.171	0.151	0.175	0.167	0.195	0.190	**0.206**	0.348
NR + GG	UG	SHTWT	0.014	0.059	0.042	**0.075**	0.027	0.066	0.037	0.071	**0.075**	0.244
UG + NR	GG	VIGOR	0.011	0.054	0.032	0.049	0.050	**0.061**	–	–	0.060	0.231
UG + NR	GG	MCMDS	0.374	0.325	0.377	0.341	0.372	0.374	–	–	**0.382**	0.558
UG + NR	GG	DM	0.216	0.173	0.221	0.212	0.235	0.238	–	–	**0.244**	0.666
UG + NR	GG	HI	**0.261**	0.210	0.252	0.204	0.222	0.213	–	–	0.215	0.386
UG + NR	GG	RTWT	0.079	0.077	**0.095**	0.073	0.084	0.061	–	–	0.063	0.320
UG + NR	GG	RTNO	**0.132**	0.096	0.130	0.110	0.113	0.097	–	–	0.099	0.345
UG + NR	GG	SHTWT	0.154	0.110	**0.163**	0.160	0.145	0.156	–	–	0.162	0.321
GG + UG	NR	VIGOR	**0.054**	-0.003	0.029	0.003	0.039	0.014	0.017	0.011	0.016	-0.024
GG + UG	NR	MCMDS	0.193	0.138	0.186	0.154	0.189	**0.190**	0.193	0.188	**0.213**	0.188
GG + UG	NR	DM	0.116	0.110	0.151	0.142	0.166	0.155	0.168	0.167	**0.184**	0.104
GG + UG	NR	HI	0.149	0.122	0.157	0.145	0.151	0.151	0.164	0.155	**0.181**	0.271
GG + UG	NR	RTWT	0.080	0.070	**0.120**	0.048	0.099	0.058	0.096	0.071	0.082	0.220
GG + UG	NR	RTNO	0.074	0.064	**0.066**	0.051	0.041	0.054	0.040	0.053	0.053	0.180
GG + UG	NR	SHTWT	0.094	0.089	0.107	0.088	0.107	0.099	0.112	0.106	**0.119**	0.169

† CVGBLUP = cross-validation GBLUP within the test population; GG, International Institute of Tropical Agriculture Genetic Gain germplasm collection; NR, National Root Crops Research Institute; UG, National Crops Resources Research Institute; DM, dry matter content; HI, harvest index; RTWT, root weight; RTNO, root number; SHTWT, shoot weight; MCMDS, mean cassava mosaic disease severity; VIGOR, early plant vigor.

For plant vigor, MCMDS, and HI, the optimized STPGA subsets gave higher predictive accuracies than the combined GG + NR full training dataset. With few exceptions (MCMDS, SHTWT, and DM), the optimized STPGA datasets gave better prediction accuracies than the same sized random sets. As the optimized STPGA dataset increased in size, the predictive accuracy did not increase, except for RTNO, where the highest predictive accuracy was found when the TP size was 1200.

When the combined GG + UG full training dataset was used to predict the NRCRI TP, Random Forest and RKHS prediction models performed better for RTWT, SHTWT, RTNO, and plant vigor. Bayesian models gave better predictive accuracies for MCMDS and DM. For plant vigor, MCMDS and DM, the combined UG+GG full dataset gave better predictive accuracies than the GBLUP cross-validation model (Supplemental Fig. S8; Supplemental Table S2b). For prediction of the NRCRI TP, the optimized STPGA selected datasets gave better predictive accuracies for plant vigor, RTWT, RTNO, and SHTWT than the combined UG+ GG full training dataset.

To predict the NRCRI TP for all traits except RTNO (at *n* = 900 and *n* = 1200) and CMD (*n* = 900), the optimized datasets gave higher predictive accuracies than the random datasets. For plant vigor, CMD resistance, and DM, the selection of optimized datasets with STPGA gave better predictive accuracies than the GBLUP cross-validation model.

Among the STPGA datasets, the highest predictive accuracy was not always the result of an increase in TP size. For CMD resistance, the highest predictive accuracy was found for the smallest optimized dataset, with the same value as the highest optimized size,.

The predictive accuracy results of traits in the GG dataset using the full training set (UG+NR) varied across methods. Whereas Bayesian methods gave better predictive accuracy values for MCMD and plant vigor, RKHS performed better for DM, HI, RTWT, and SHTWT. The combined (UG+NR) full training dataset for predicting the GG population gave lower predictive accuracies than the GBLUP cross-validation model for all traits. The GBLUP cross-validation model also gave better predictive accuracies for all the traits than the random and optimized STPGA datasets. The optimized STPGA datasets gave better predictive accuracies than the random sets for all traits except for plant vigor and for DM (optimized dataset *n* = 900) (Supplemental Fig. S9; Supplemental Table S2b). For all traits except MCMDS and DM, the optimized STPGA subsets gave higher predictive accuracies than the combined UG+NR full training dataset.

For all the cross-population results, we tested if the optimized STPGA sets would do better than random with a binomial test, assuming the independence of the comparisons. We compared how many times the prediction accuracy of STPGA was greater than random for all traits. We found that for prediction of the NR and UG sets, the STPGA-optimized sets performed better than the random sets. On the contrary, when we applied the same comparison of the STPGA sets with the predictions with full sets, the latter had a significantly higher number of full sets that was greater than STPGA’s predictive accuracy results.

Additionally, we tested if there was differential enrichment in the optimized STPGA training set of any of the populations relative to the source sets. We found a significant enrichment of the GG population (*p* < 0.001) in the STPGA of different sizes for the prediction of the NR set with GG + UG. Similarly, we found a significant enrichment of the NR population (*p* < 0.001) in the STPGA of different sizes for predicting the GG set with the UG-NR. On the contrary, we found no significant enrichment of any population in the STPGA-optimized sets predicting the UG population.

### Cross-Generation Prediction

**Fig. 5:**
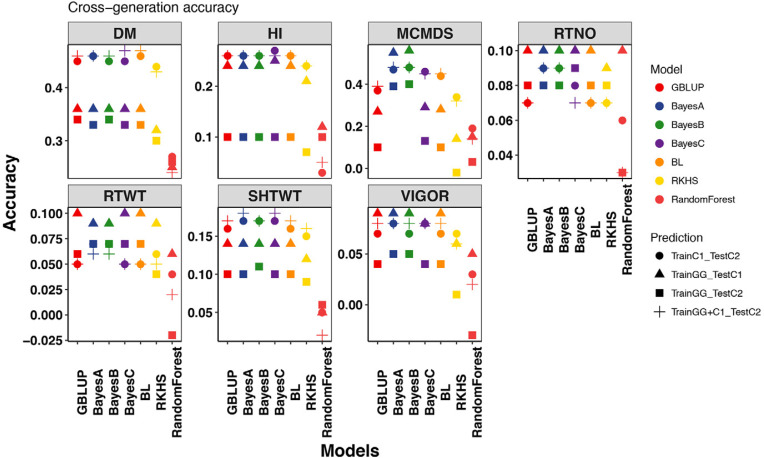
Plot of cross-generation prediction accuracies. Seven genomic prediction methods were tested for seven traits (panels). For each model (colors, *x*-axis within panels), four predictions were made: Genetic Gain (GG) predicting Cycle 1 (C1), GG predicting (Cycle 2) C2, C1 predicting C2, and GG + C1 predicting C2, indicated by shapes. All data are from the International Institute for Tropical Agriculture (IITA) genomic selection program. DM, dry matter content; HI, harvest index; RTWT, root weight; RTNO, root number; SHTWT, shoot weight; MCMDS, mean cassava mosaic disease severity; VIGOR, early plant vigor.

One major area where analysis was needed concerned prediction across generations. Selections can be done at the seedling stage if GEBV can be predicted from the previous generations and training data. Because nearly all of the IITA germplasm from C1 and C2 were clonally evaluated, we were able to use these data to assess the accuracy of genomic predictions on unevaluated genotypes of the next generation. In general, the accuracy of prediction across generations was greatest when predicting C2, as shown by averaging across prediction models and traits for predictions trained either with C1 (mean = 0.19 ± SE 0.02) or GG + C1 (0.19 ± 0.02). The accuracy was lower on average when we predicted C2 with GG (0.11 ± 0.01) than when we predicted C1 with GG (0.17 ± 0.02). Accuracy was lowest for both plant vigor and RTWT (0.06 ± 0.005) and was highest for MCMDS (0.32 ± 0.03) and DM (0.38 ± 0.01). Most prediction models performed similarly, as shown by the averaged accuracy across traits and training–test combinations, with RF performing worst (0.08 ± 0.01) and BayesA and BayesB performing best (both 0.20 ± 0.03). For MCMDS, we found that prediction accuracy was greatest with BayesA and BayesB (Fig. 5, Supplemental Fig. S10, Supplemental Table S3).

### Training Population Update

The first 100 principal components of the C1 kinship matrix were used as predictors for STPGA and explained 97.7% of the genetic variance. In all cases, the genetic algorithm converged within the 1000-iteration run (Supplemental Fig. S11).

Given the constraints of breeding programs described above, it was necessary to select samples of C1 that were optimized for predicting the parents of C2, rather than the C2 themselves. Despite targeting the parents of C2, we used selected training sets to predict C2, thus simulating the addition of phenotypes to the training set. Because of this, we compared the accuracy of subsets of C1 predicting C2 to the accuracy of predicting the parents of C2. As the number sampled increased from 200 to 2400, averaging across traits and methods for subset selection (STPGA and at random), accuracy increased by 120 and 105% when predicting C2 and the parents of C2, respectively. The increase in accuracy was smaller when we included the 709 GG clones in the prediction, increasing only by 43 and 36% respectively when predicting C2 and parents of C2 (Supplementary Table S4).

The STPGA approach consistently selected training datasets with a lower expected mean PEV on the test set than random sampling, across training set sizes (Supplemental Fig. S12). Further, using STPGA to select clones for phenotyping gave 13% better accuracy on average (average accuracy of 0.242 vs. 0.214, two-tailed *t* = 6.29, df = 4458, *p* < 0.0001) than random sampling. Broken down by validation set, STPGA was significantly better than random for predicting the parents of C2 (*t* = 9.8, df = 2147, *p* < 0.0001) but was not significantly better for predicting C2 (*t* = 1.41, df = 2227, *p* = 0.16).

**Fig. 6:**
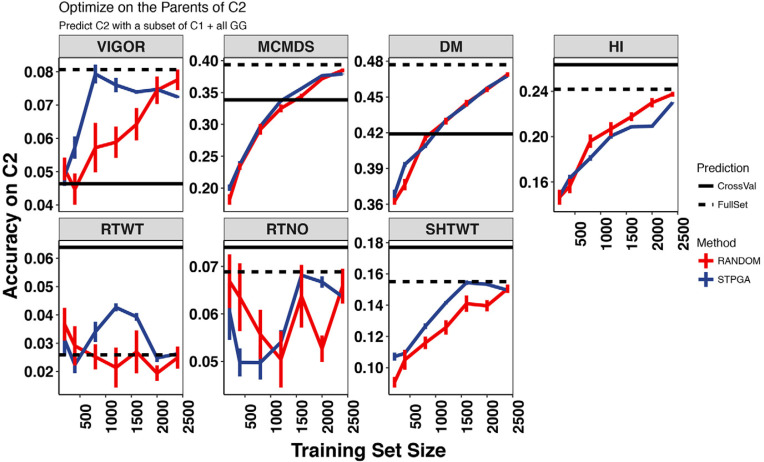
The relationship between training set size and the accuracy of predicting the International Institute for Tropical Agriculture Cycle 2 (C2) (across generations). The accuracy of prediction for seven traits (panels) with the IITA Genetic Gain (GG) population training data plus data from different sized subsets (*x*-axis) of their progeny, Cycle 1 (C1) is shown. Subsets of a given size were selected either at random or with the genetic algorithm implemented in the R package STPGA. Ten random and 10 STPGA-selected subsets were made for each training set size. Error bars are the SE around the mean for the ten samples. Horizontal black lines show the mean crossvalidation accuracy for C2 (validation set; solid line) and the accuracy of the full set of GG + C1 predicting C2 (dashed line). DM, dry matter content; HI, harvest index; RTWT, root weight; RTNO, root number; SHTWT, shoot weight; MCMDS, mean cassava mosaic disease severity; VIGOR, early plant vigor.

**Fig. 7:**
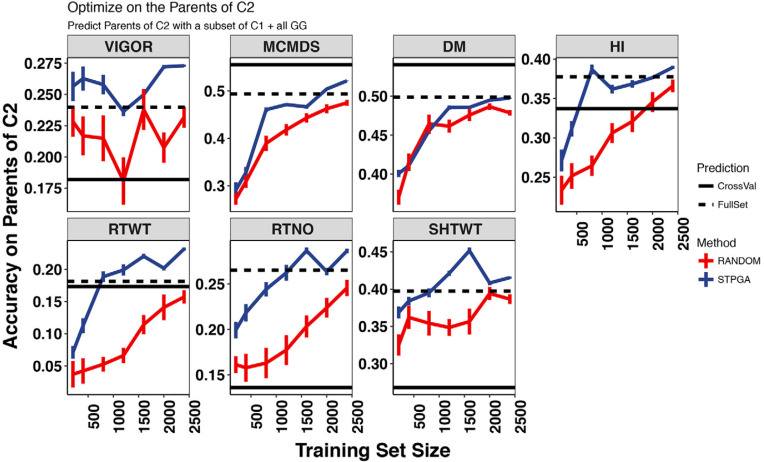
The relationship between training set size and the accuracy of predicting the parents of Cycle 2 (C2) [from Cycle 1 (C1), withingeneration). The accuracy of the predictions for seven traits (panels) with the International Institute for Tropical Agriculture Genetic Gain (GG) population training data plus data from different sized subsets (*x*-axis) of their progeny, Cycle 1 is shown. Subsets of a given size were selected either at random or with the genetic algorithm implemented in the R package STPGA. Ten random and 10 STPGAselected subsets were made for each training set size. Error bars are the SE around the mean for the 10 samples. Horizontal black lines show the mean cross-validation accuracy for C1 (validation set; solid line) and the accuracy of the full set of GG + C1 predicting the parents of C2 (dashed line). DM, dry matter content; HI, harvest index; RTWT, root weight; RTNO, root number; SHTWT, shoot weight; MCMDS, mean cassava mosaic disease severity; VIGOR, early plant vigor.

We compared these accuracies with that of the full set of C1 (or GG + C1) and to the cross-validation accuracy within the test set (C1 for prediction of the parents of C2, and C2 for predictions of C2). When predicting C2, which was our primary goal, the subsets were almost always inferior to the full set, with the exceptions of the middle sizes for RTWT, but the advantage was very small (Fig. 6, Supplemental Fig. S13). However, STPGA-selected subsets tended to have better accuracy than the full set, especially for yield components when predicting the parents of C2, which were the genotypes targeted by the optimization algorithm (Fig. 7, Supplemental Fig. S14).

The correlation between the selection criterion (mean PEV) used by STPGA and the training set size is strong for all traits (range = –0.57 to –0.61). Aside from simply increasing the TP size, we wanted to assess the extent to which the mean PEV could be used as a predictor of the achievable accuracy. Regression of prediction accuracies for each sample (regardless of whether it was selected randomly or by STPGA) on mean PEV explained between 8% (RTNO) and 46% (DM) of the variance in accuracy. Multiple regression including mean PEV and training set size as predictors showed PEV to be the more significant predictor (across all traits). In fact, training set size was not a significant explanatory variable for RTWT or RTNO (Supplemental Table S5).

## Discussion

The Next Generation Cassava Breeding Project (www.nextgencassava.org, accessed 15 Aug. 2017) aims to assess the potential of genomic selection in cassava to reduce the length of the breeding cycle and increase the number of crosses and selections per unit of time. The project is implementing GS in three breeding programs from Nigeria and Uganda, with genotypic and phenotypic data from TPs and two cycles of selection available on a database dedicated to cassava (www.cassavabase.org, accessed 15 Aug. 2017).

Using a cross-validation scheme, we contrasted the performance of GBLUP, RKHS (single-kernel and multikernel), BayesA, BayesB, BayesCpi, Bayesian LASSO, and RF for yield components (RTWT, RTNO, SHTWT, HI, and DM) and CMD resistance data from the breeding programs.

In general, the performance of predictive models is known to be conditional on the genetic architecture of the trait under consideration (Daetwyler et al., 2010; Su et al., 2014). Although nonadditive models, including RF and RKHS, capture dominance and epistasis effects, GBLUP is more suitable for prediction when traits are determined by an infinite number of unlinked and nonepistatic loci, with small effects.

Not surprisingly, heritability varied between populations, conceivably as a consequence of the differences in the number and design of field trials among breeding programs. For most traits, it is not possible to determine the reason for differences in heritability exactly. However, for DM, we can hypothesize that the difference in phenotyping protocols between programs (the specific gravity method at NRCRI and NaCRRI versus oven drying at IITA) could account for the observed differences. We note the estimate of zero heritability for RTWT, RTNO, and SHTWT in the IITA C2 and acknowledge this is likely to account for the quality of cross-generation prediction in that dataset.

The cross-validation results were mostly consistent across breeding programs and the superiority of one prediction method over the others was trait-dependent. Random Forest and RKHS usually predicted phenotypes more accurately for yield-related traits, which are known to have a significant amount of nonadditive genetic variation (Wolfe et al., 2016a). Similar findings have been made in wheat (*Triticum aestivum* L.) for grain yield, an additive and epistatic trait, in which RKHS, radial basis function neural networks, and Bayesian regularized neural networks models clearly had a better predictive ability than additive models like BL, Bayesian ridge-regression, BayesA, and BayesB (Perez-Rodriguez et al., 2013).

Though the cross-validation results within the breeding programs are encouraging for the use of GS, prediction values across breeding programs were fairly low. Mean F_ST_ values were low (less than 0.05), indicating that the three breeding populations share genetic material. Despite this, our results indicate that the prospect for sharing data across Africa to assist in GS is limited to certain traits (most notably MCMDS) and populations. Indeed, obtaining a larger training set by combining TP did not always lead to higher prediction accuracies than what could already be achieved within that population, as shown by the cross-validation results.

In animal models, prediction with multibreed populations has also been shown to be poor, with most of the observed accuracy caused by population structure (Daetwyler et al., 2012). An alternative kernel function has been proposed to estimate the covariance between individuals based on markers, which can improve the fit to the data to account for the genetic heterogeneity of breeding populations (Heslot and Jannink, 2015).

Conceivably, in our study, the addition of individuals from different breeding programs was detrimental caused by the inconsistent heritability of most traits. Another possibility is genotype × environment interaction. The impact of genotype × environment interactions on predictive accuracy has been reported in wheat when the same population was evaluated in different environments (Crossa et al., 2010; Endelman, 2011). Similarly, in cassava with historical data from the IITA’s GG population, prediction across locations led to a decrease in accuracy (Ly et al., 2013).

Using the training sets selected based on an optimized algorithm gave better predictive ability than randomly assigned samples but showed a decrease in accuracy when compared with the GBLUP cross-validation results. Although in previous studies, the predictive accuracies with full sets were lower than those obtained with optimized subsets (Rutkoski et al., 2015), in our study, we found the opposite, indicating that a larger training set was more advantageous. Combining data from different experiments and populations for cross-population prediction remains promising for traits like CMD, where the GWAS results indicate a stable large-effect quantitative trait loci throughout the tested breeding populations (Wolfe et al., 2016b).

When predicting unevaluated progenies from the next generation (cross-generation prediction), our results indicated, in our judgment, that accuracy should be sufficient for DM, MCMDS, and, to a lesser extent, HI (Fig. 5). Although accuracy is stable across the generations tested for DM with most models, for MCMDS to be successful, we recommend using a Bayesian shrinkage model such as BayesA or BayesB. The advantage of these models over GBLUP for CMD resistance probably arises because of the major known quantitative trait loci segregating in the population (Rabbi et al., 2014; Wolfe et al., 2016a) and the ability of these two models to allow differential contributions of markers near the quantitative trait loci to the prediction. One disadvantage of BayesB, in particular, is that the known polygenic background resistance for CMD may become deemphasized in favor of heavy selection on the major effect gene(s) (Hahn et al., 1980; Legg and Thresh, 2000; Akano et al., 2002; Rabbi et al., 2014; Wolfe et al., 2016b).

We noted that RF and RKHS performed poorly across generations; this is a result that makes sense, given that the predictability of epistatic and dominant interactions declines with recombination (Lynch and Walsh, 1998).

On the basis of the datasets analyzed in this study, it was apparent that the size of a TP had a significant impact on prediction accuracy for most traits. Thus breeding programs will benefit from phenotyping the maximum possible amount. In agreement with the results in other crops (Rincent et al., 2012; Akdemir et al., 2015; Isidro et al., 2015), our results indicate that optimization algorithms like STPGA can provide at least a small advantage over random selection of materials for phenotyping.

Each breeding program will need to determine the amount of phenotyping vs. genotyping to do to maximize prediction accuracy and selection gain based on the cost and availability of land, labor, and genotyping. An analysis in barley (*Hordeum vulgare* L.) by Endelman et al. (2014) provides a good example of the potential complexity of these decisions. The authors show, as we do, that having a larger number of phenotyped individuals is always beneficial, and that it is usually beneficial to focus on evaluating new lines at the expense of additional phenotyping of old lines. However, if genotyping costs are high, the cost–benefit balance shifts toward more evaluation of the existing lines (Endelman et al., 2014). Endelman et al.’s (2014) study focused on prediction in biparental populations. Although this is likely to apply to cassava breeding populations, we stress the necessity of doing such an analysis for each breeding application separately.

An important result is that STPGA was able to find subsets that were better than the full set for predicting the parents of C2. The parents of C2 are members of C1 and were the individuals targeted by STPGA. One possible interpretation is that the benefit comes from phenotyping members of the same generation. If that were true, we could make a significant difference in accuracy by phenotyping a subset of clones from the current generation before predicting GEBV for the entire set of selection candidates. To do this without lengthening the selection and recombination cycle, harvested stems would need to be stored long enough for phenotypic data to be curated, predictions and selections to be conducted, and STPGA to be run. Methods of storing cassava stakes for up to 30 d are available, indicating that such a scheme could be possible (Sungthongw et al., 2016). Even without improved stem cutting storage, this could be done while only lengthening the selection and recombination cycle to perhaps 1.5 to 2 yr, which would still be significantly faster than conventional cassava breeding.

A related possibility is to place annual selection pressure on traits that are predictable across generation (e.g., MCMDS, HI, and DM). Predictions of total genetic value for yield traits for selection of clones that will be tested as potential varieties could then be done after clonal evaluation data become available on at least a subset of contemporary genotypes. Further trials will be necessary to determine whether there is an advantage to this type of strategy.

The primary promise GS offers to cassava breeding is the ability to select and recombine germplasm more frequently and thus hopefully speed the rate of population improvement while combining a myriad of quality, disease, and yield-related traits into a single genotype that can be released as a variety. The applicability of the results from the different prediction models in cassava is then dependent on whether the goal is the prediction of breeding values of progeny or the selection of advanced lines for testing as varieties.

We are still in the early stages of GS in this crop, but the results are promising, at least for some traits. The TPs need to continue to grow and quality phenotyping is more critical than ever. However, general guidelines for successful GS are emerging. Phenotyping can be done on fewer individuals, cleverly selected, making for trials that are more focused on the quality of the data collected.

## Supplemental Information

Supplemental File S1: Supplementary methods describing the details of the field trial design.

Supplemental Table S1: Details of the prediction scenarios tested using different sized subsets of the IITA Cycle 1.

Supplemental Table S2: Cross-population prediction results. (A) Cross-population results of seven prediction models for the combined datasets (full set model). CVGBLUP, cross-validation GBLUP results within training populations. Bold italic typeface indicates the highest predictive accuracy across prediction models.(B) Crosspopulation results for STPGA-optimized and at random with the GBLUP model. GG, IITA genetic gain; NR, National Root Crops Research Institute; UG, National Crops Resources Research Institute; DM, dry matter content; HI, harvest index; RTWT, root weight; RTNO, root number; SHTWT, shoot weight; MCMDS, mean cassava mosaic disease severity; VIGOR, early plant vigor.

Supplemental Table S3: Raw accuracies from cross-generation predictions. Accuracy levels from each combination of training set and validation set tested. Data from each trait are given in the rows and those for each model are shown in the columns.

Supplemental Table S4: Mean and standard error accuracies and PEV means for seven traits are shown. There were four combinations of training and test sets and two different subset selection methods (random vs. STPGA) considered. DM, dry matter content; HI, harvest index; RTWT, root weight; RTNO, root number; SHTWT, shoot weight; MCMDS, mean cassava mosaic disease severity; VIGOR, early plant vigor; C1, Cycle 1; C2, Cycle 2; PofC2, parents of C2.

Supplemental Table S5: The results from two regressions are shown. The top table shows the results from a multiple regression in which the mean PEV and the training set size were used as competing predictors of the accuracy achieved by different sized subsets for each trait respectively. The bottom table is a similar regression with only a single predictor, the mean PEV. In the multiple regression, predictors were mean-centered and scaled to unit variance.

Supplemental Fig. S1: Population genetic structure of the datasets analyzed in this study, illustrated by plotting the first two components (PCs), following a principal component analysis (PCA) of the genetic relationship matrix. The training populations for each breeding institute’s genomic selection program are compared on the left (A). The breeding cycles (genetic gain, Cycle 1 and Cycle 2) from the IITA genomic selection program are contrasted on the right (B).

Supplemental Fig. S2: Histogram of the kinship coefficients [off-diagonals of the genomic relationship matrix (GRM)] on left and inbreeding values (diagonals of the GRM) on the right for all five datasets analyzed in this study. The GRM was constructed for each dataset separately with markers with >1% minor allele frequency. IITA, International Institute of Tropical Agriculture; GG, IITA genetic gain; C1, IITA Cycle 1; C2, IITA Cycle 2; NR, National Root Crops Research Institute; UG, National Crops Research Resources Institute.

Supplemental Fig. S3: GS cross-validation accuracies in the NaCRRI dataset. Fivefold cross-validation results are shown for seven traits measured with GBLUP, RKHS (GAUSS_K0.01, single RKHS kernel; MultiKernelGauss, and multikernel RKHS), BayesA, BayesB, BayesC, Bayesian Lasso (BL), and Random Forest. DM, dry matter content; HI, harvest index; RTWT, root weight; RTNO, root number; SHTWT, shoot weight; MCMDS, mean cassava mosaic disease severity; VIGOR, early plant vigor.

Supplemental Fig. S4: GS cross-validation accuracies in the NRCRI dataset. Fivefold cross-validation results are shown for seven traits with GBLUP, RKHS (GAUSS_K0.01, single RKHS kernel; MultiKernelGauss, multikernel RKHS), BayesA, BayesB, BayesC, Bayesian Lasso (BL), and Random Forest. DM, dry matter content; HI, harvest index; RTWT, root weight; RTNO, root number; SHTWT, shoot weight; MCMDS, mean cassava mosaic disease severity; VIGOR, early plant vigor.

Supplemental Fig. S5: GS cross-validation accuracies in the Genetic Gain dataset. Fivefold cross-validation results are shown for seven traits with GBLUP, RKHS (GAUSS_K0.01, single RKHS kernel; MultiKernelGauss, multikernel RKHS), BayesA, BayesB, BayesC, Bayesian Lasso (BL), and Random Forest. DM, dry matter content; HI, harvest index; RTWT, root weight; RTNO, root number; SHTWT, shoot weight; MCMDS, mean cassava mosaic disease severity; VIGOR, early plant vigor.

Supplemental Fig. S6: Hierarchical clustering of genomic prediction models based on the cross-validated genomic estimated breeding values (GEBVs). Height on the *y*-axis refers to the value of the dissimilarity criterion. Clustering of the prediction models in the combined results for all populations is shown. GBLUP, genomic best linear unbiased predictor; BL, Bayesian Lasso; RF, Random Forest; RKHS, reproducing kernel Hilbert space (multikernel model).

Supplemental Fig. S7: Cross-population prediction of UG, showing the accuracy of the predictions for seven traits with the combined NR + GG population training data. Subset sizes (*x*-axis) were selected either at random or by using the genetic algorithm implemented in the R package STPGA. Ten random and 10 STPGA-selected subsets were made at each training set size. Error bars are the SE around the mean for the 10 samples. Horizontal lines show the mean cross-validation accuracy for the UG population (validation set, orange line) and the accuracy of the full NR + GG set predicting the UG population (red line).

Supplemental Fig. S8: Cross-population prediction of NR, showing the accuracy of the predictions for seven traits with the combined UG + GG population training data. Subsets sizes (*x*-axis) were selected either at random or by using the genetic algorithm implemented in the R package STPGA. Ten random and 10 STPGA-selected subsets were made at each training set size. Error bars are the SE around the mean for the 10 samples. Horizontal lines show the mean cross-validation accuracy for the NRCRI population (validation set, orange line) and the accuracy of the full UG + GG set predicting the NR population (red line).

Supplemental Fig. S9: Cross-population prediction of GG, showing the accuracy of prediction for seven traits with the combined NR + UG population training data. Subset sizes (*x*-axis) were selected either at random or by using the genetic algorithm implemented in the R package STPGA. Ten random and 10 STPGA-selected subsets were made at each training set size. Error bars are the SE around the mean for the 10 samples. Horizontal lines show the mean cross-validation accuracy for the GG population (validation set, orange line) and the accuracy of the full NR + UG set predicting the GG population (red line).

Supplemental Fig. S10: Cross-generation prediction accuracies. In the IITA genomic selection dataset, there are three generations of clones: the genetic gain (GG), their progeny in Cycle 1 (C1), and C1’s progeny, Cycle 2 (C2). For each of seven traits (rows) and seven prediction models (*x*-axis, colors), we made four cross-generation predictions (columns): GG predicting C1, GG predicting C2, C1 predicting C2, and GG + C1 predicting C2. DM, dry matter content; HI, harvest index; RTWT, root weight; RTNO, root number; SHTWT, shoot weight; MCMDS, mean cassava mosaic disease severity; VIGOR, early plant vigor.

Supplemental Fig. S11: Convergence of the genetic algorithm implemented in the R package STPGA. Plot of the optimization criterion (mean PEV, *y*-axis) versus the iteration of the genetic algorithm (*x*-axis) across training sample sizes (panels). Samples were drawn from the IITA Cycle 1 (C1), excluding the parents of Cycle 2 (PofC2). The algorithm was set to find the smallest mean PEV with the PofC2 as the test (validation) set and a sample of C1 as the training set. Ten runs of the genetic algorithm are shown in different colored lines.

Supplemental Fig. S12: Does STPGA find lower mean PEV across sample sizes than random selection? The size of training samples used in four prediction scenarios (rows) is plotted against the mean PEV of the subset for every trait and each of 10 samples elected either by the genetic algorithm implemented in the R package STPGA (red) or randomly (blue). The actual mean PEV and number of training samples are plotted here, with variations from planned sample sizes and PEV means initially expected because of missing data for some traits or individuals. The genetic algorithm implemented by STPGA was run 10 times. The validation set target for the optimization algorithm were the parents of IITA’s Cycle 2 (PofC2) and the training sets were samples of differing size of the IITA Cycle 1 (C1). Predictions were made either with samples of C1 only (Rows 1 and 2) or with samples of C1 plus the entire GG (Rows 3 and 4). Validation sets were either the PofC2 (Rows 1 and 3) or the C2 (Rows 2 and 4).

Supplemental Fig. S13: The relationship between training set size and accuracy predicting IITA Cycle 2 (across generations). The accuracy of prediction for seven traits (panels) with different sized subsets (*x*-axis) of IITA Cycle 1 (C1) is shown. Subsets of a given size were selected either at random or with the genetic algorithm implemented in the R package STPGA. Ten random and 10 STPGA-selected subsets were made at each training set size. Error bars are the SE around the mean for the 10 samples. Horizontal black lines show the mean cross-validation accuracy for Cycle 2 (C2, validation set; solid line) and the accuracy of the full set of GG + C1 predicting C2 (dashed line). GBLUP was used for all predictions. DM, dry matter content; HI, harvest index; RTWT, root weight; RTNO, root number; SHTWT, shoot weight; MCMDS, mean cassava mosaic disease severity; VIGOR, early plant vigor.

Supplemental Fig. S14: The relationship between training set size and the accuracy of predicting the parents of Cycle 2 (C2) from Cycle 1 (C1) (within-generation prediction). The accuracy of prediction for seven traits (panels) with different sized subsets (*x*-axis) of the IITA C1 is shown. Subsets of a given size were selected either at random or with the genetic algorithm implemented in the R package STPGA. Ten random and 10 STPGAselected subsets were made at each training set size. Error bars are the SE around the mean for the 10 samples. Horizontal black lines show the mean crossvalidation accuracy for the C1 (validation set; solid line) and the accuracy of the full set of GG + C1 predicting the parents of C2 (dashed line). GBLUP was used for all predictions. DM, dry matter content; HI, harvest index; RTWT, root weight; RTNO, root number; SHTWT, shoot weight; MCMDS, mean cassava mosaic disease severity; VIGOR, early plant vigor.

## References

[cit0001] Akano O., Dixon O, Mba C, Barrera E, and Fregene M 2002 Genetic mapping of a dominant gene conferring resistance to cassava mosaic disease. Theor. Appl. Genet. 105(4):521–525. doi:10.1007/s00122-002-0891-7 12582500 10.1007/s00122-002-0891-7

[cit0002] Akdemir D., and Okeke U.G 2015 EMMREML: Fitting mixed models with known covariance structures. https://CRAN.R-project.org/package=EMMREML (accessed 30 Aug. 2017).

[cit0003] Akdemir D., Sanchez J.I, and Jannink J.-L 2015 Optimization of genomic selection training populations with a genetic algorithm. Genet. Sel. Evol. 47(1):38. doi:10.1186/s12711-015-0116-6 25943105 10.1186/s12711-015-0116-6 PMC4422310

[cit0004] Bamidele O.P., Fasogbon M.B, Oladiran D.A, and Akande E.O 2015 Nutritional composition of fufu analog flour produced from Cassava root (Manihot esculenta) and Cocoyam (Colocasia esculenta) tuber. Food Sci. Nutr. 3(6):597–603. doi:10.1002/fsn3.250 26788301 10.1002/fsn3.250 PMC4708653

[cit0005] Barabaschi D., Tondelli A, Desiderio F, Volante A, Vaccino P, Vale G, et al 2015 Next generation breeding. Plant Sci. 242:3–13. doi:10.1016/j.plantsci.2015.07.010 26566820 10.1016/j.plantsci.2015.07.010

[cit0006] Bates D., Maechler M, Bolker B, and Walker S 2014 Fitting linear mixed-effects models using lme4. J. Stat. Softw. 67(1):1–48.

[cit0007] Breiman L 2001 Random forests. Mach. Learn. 45(1):5–32. doi:10.1023/A:1010933404324

[cit0008] Browning B.L., and Browning S.R, 2009 A unified approach to genotype imputation and haplotype-phase inference for large data sets of trios and unrelated individuals. Am. J. Hum. Genet. 84:210–223. 10.1016/j.ajhg.2009.01.005 PMC2668004 19200528

[cit0009] Ceballos H., Kawuki R.S, Gracen V.E, Yencho G.C, and Hershey C.H 2015 Conventional breeding, marker-assisted selection, genomic selection and inbreeding in clonally propagated crops: A case study for cassava. Theor. Appl. Genet. doi:10.1007/s00122-015-2555-4 10.1007/s00122-015-2555-4 PMC4540783 26093610

[cit0010] Charmet G., Storlie E, Oury F.X, Laurent V, Beghin D, Chevarin L, et al 2014 Genome-wide prediction of three important traits in bread wheat. Mol. Breed. 34(4):1843–1852. doi:10.1007/s11032-014-0143-y 26316839 10.1007/s11032-014-0143-y PMC4544631

[cit0011] Chukwuemeka O.C 2007 Effect of process modification on the physiochemical and sensory quality of fufu-flour and dough. Afr. J. Biotechnol. 6(8):1949–1953.

[cit0012] Cros D., Denis M, Sánchez L, Cochard B, Flori A, Durand-Gasselin T, et al 2015 Genomic selection prediction accuracy in a perennial crop: Case study of oil palm (Elaeis guineensis Jacq.). Theor. Appl. Genet. 128(3):397–410. doi:10.1007/s00122-014-2439-z 25488416 10.1007/s00122-014-2439-z

[cit0013] Crossa J., de los Campos G, Perez P, Gianola D, Burgueno J, Araus J.L, et al 2010 Prediction of genetic values of quantitative traits in plant breeding using pedigree and molecular markers. Genetics 186(2):713–724. doi:10.1534/genetics.110.118521 20813882 10.1534/genetics.110.118521 PMC2954475

[cit0014] Daetwyler H.D., Kemper K.E, van der Werf J.H, and Hayes B.J 2012 Components of the accuracy of genomic prediction in a multi-breed sheep population. J. Anim. Sci. 90:3375–3384. doi:10.2527/jas.2011-4557 23038744 10.2527/jas.2011-4557

[cit0015] Daetwyler H.D., Pong-Wong R, Villanueva B, and Woolliams J.A 2010 The impact of genetic architecture on genome-wide evaluation methods. Genetics 185(3):1021–1031. doi:10.1534/genetics.110.116855 20407128 10.1534/genetics.110.116855 PMC2907189

[cit0016] Danecek P., Auton A, Abecasis G, Albers C.A, Banks E, DePristo M.A, et al 2011 The variant call format and VCFtools. Bioinformatics 27(15):2156–2158. doi:10.1093/bioinformatics/btr330 21653522 10.1093/bioinformatics/btr330 PMC3137218

[cit0017] Dekkers J.C.M., and Hospital F 2002 The use of molecular genetics in the improvement of agricultural populations. Nat. Rev. Genet. 3(1):22–32. doi:10.1038/nrg701 11823788 10.1038/nrg701

[cit0018] Elshire R.J., Glaubitz J.C, Sun Q, Poland J.A, Kawamoto K, Buckler E.S, et al 2011 A robust, simple genotyping-by-sequencing (GBS) approach for high diversity species. PLoS One 6(5):E19379. doi:10.1371/journal.pone.0019379 21573248 10.1371/journal.pone.0019379 PMC3087801

[cit0019] Endelman J.B 2011 Ridge Regression and Other Kernels for Genomic Selection with R Package rrBLUP. Plant Genome 4(3):250. doi:10.3835/plantgenome2011.08.0024

[cit0020] Endelman J.B., Atlin G.N, Beyene Y, Semagn K, Zhang X, Sorrells M.E, et al 2014 Optimal design of preliminary yield trials with genomewide markers. Crop Sci. 54(1):48–59. doi:10.2135/cropsci2013.03.0154

[cit0021] Esuma W., Herselman L, Labuschagne M.T, Ramu P, Lu F, Baguma Y, et al ,2016 Genome-wide association mapping of provitamin A carotenoid content in cassava. Euphytica. doi:10.1007/s10681-016-1772-5

[cit0022] Garrick D.J., Taylor J.F, and Fernando R.L 2009 Deregressing estimated breeding values and weighting information for genomic regression analyses. Genet. Sel. Evol. 41:55. doi:10.1186/1297-9686-41-55 20043827 10.1186/1297-9686-41-55 PMC2817680

[cit0023] Gianola D., and van Kaam J.B.C.H.M 2008 Reproducing kernel Hilbert spaces regression methods for genomic assisted prediction of quantitative traits. Genetics 178(4):2289–2303. doi:10.1534/genetics.107.084285 18430950 10.1534/genetics.107.084285 PMC2323816

[cit0024] Gianola D., de los Campos G, Hill W.G, Manfredi E, and Fernando R 2009 Additive genetic variability and the Bayesian alphabet. Genetics 183(1):347–363. doi:10.1534/genetics.109.103952 19620397 10.1534/genetics.109.103952 PMC2746159

[cit0025] Glaubitz J.C., Casstevens T.M, Lu F, Harriman J, Elshire R.J, Sun Q, et al 2014 TASSEL-GBS: A high capacity genotyping by sequencing analysis pipeline. PLoS One 9(2):E90346. doi:10.1371/journal.pone.0090346 24587335 10.1371/journal.pone.0090346 PMC3938676

[cit0026] Gouy M., Rousselle Y, Bastianelli D, Lecomte P, Bonnal L, Roques D, et al 2013 Experimental assessment of the accuracy of genomic selection in sugarcane. Theor. Appl. Genet. 126:2575–2586. doi:10.1007/s00122-013-2156-z 23907359 10.1007/s00122-013-2156-z

[cit0027] Habier D., Fernando R.L, Kizilkaya K, and Garrick D.J 2011 Extension of the Bayesian alphabet for genomic selection. BMC Bioinformatics 12(1):186. doi:10.1186/1471-2105-12-186 21605355 10.1186/1471-2105-12-186 PMC3144464

[cit0028] Hahn S., Howland A, and Terry E 1980 Correlated resistance of cassava to mosaic and bacterial blight diseases. Euphytica 29:305–311. doi:10.1007/BF00025127

[cit0029] Hamblin M.T., and Rabbi I.Y 2014 The effects of restriction-enzyme choice on properties of genotyping-by-sequencing libraries: A study in cassava. Crop Sci. 54(6):2603. doi:10.2135/cropsci2014.02.0160

[cit0030] Heffner E.L., Sorrells M.E, and Jannink J.-L 2009 Genomic selection for crop improvement. Crop Sci. 49(1):1. doi:10.2135/cropsci2008.08.0512

[cit0031] Heslot N., and Jannink J.-L 2015 An alternative covariance estimator to investigate genetic heterogeneity in populations. Genet. Sel. Evol. 47(1):93. doi:10.1186/s12711-015-0171-z 26612537 10.1186/s12711-015-0171-z PMC4661961

[cit0032] Heslot N., Yang H.-P, Sorrells M.E, and Jannink J.-L 2012 Genomic selection in plant breeding: A comparison of models. Crop Sci. 52(1):146–160. doi:10.2135/cropsci2011.06.0297

[cit0033] Howeler R., Lutaladio N, and Thomas G 2013 Save and grow: Cassava. A guide to sustainable production intensification. Food and Agriculture Organization of the United Nations, Rome.

[cit0034] International Cassava Genetic Map Consortium 2015 High-resolution linkage map and chromosome-scale genome assembly for cassava (Manihot esculenta Crantz) from ten populations. G3 (Bethesda) 5(1):133–144. 10.1534/g3.114.015008 PMC4291464 25504737

[cit0035] Isidro J., Jannink J.-L, Akdemir D, Poland J, Heslot N, and Sorrells M.E 2015 Training set optimization under population structure in genomic selection. Theor. Appl. Genet. 128:145–158. doi:10.1007/s00122-014-2418-4 25367380 10.1007/s00122-014-2418-4 PMC4282691

[cit0036] Jannink J.-L 2010 Dynamics of long-term genomic selection. Genet. Sel. Evol. 42:35. doi:10.1186/1297-9686-42-35 20712894 10.1186/1297-9686-42-35 PMC2936280

[cit0037] Kawano K., Fukuda W.M.G, and Cenpukdee U 1987 Genetic and environmental effects on dry matter content of Cassava root. Crop Sci. 27(1):69. doi:10.2135/cropsci1987.0011183X002700010018x

[cit0038] Kawano K., Narintaraporn K, Narintaraporn P, Sarakarn S, Limsila A, Limsila J, et al 1998 Yield improvement in a multistage breeding program for cassava. Crop Sci. 38: 325–332.

[cit0039] Kohavi R 1995 A study of cross-validation and bootstrap for accuracy estimation and model selection. Int. Joint Conf. on Artificial Intelligence 14(12): 1137–1143.

[cit0040] Legarra A., Robert-Granié C, Croiseau P, Guillaume F, and Fritz S 2011 Improved Lasso for genomic selection. Genet. Res. 93(1):77–87. doi:10.1017/S0016672310000534 10.1017/S0016672310000534 21144129

[cit0041] Legg J.P., and Thresh J.M 2000 Cassava mosaic virus disease in East Africa: A dynamic disease in a changing environment. Virus Res. 71(1–2):135–149. doi:10.1016/S0168-1702(00)00194-5 11137168 10.1016/s0168-1702(00)00194-5

[cit0042] Liaw A., and Wiener M 2002 Classification and regression by random-Forest. R News 2(12): 18–22.

[cit0043] Lorenz A.J., Chao S, Asoro F.G, Heffner E.L, Hayashi T, Iwata H, et al 2011 Genomic selection in plant breeding: Knowledge and prospects. In: Sparks D.L., editor, Advances in Agronomy, Vol. 110 Academic Press, Cambridge, MA.

[cit0044] Ly D., Hamblin M, Rabbi I, Melaku G, Bakare M, Gauch H.G, et al 2013 Relatedness and genotype × environment interaction affect prediction accuracies in genomic selection: A study in cassava. Crop Sci. 53(4):1312. doi:10.2135/cropsci2012.11.0653

[cit0045] Lynch M., and Walsh B 1998 Genetics and analysis of quantitative traits. Sinauer Associates, Inc, Sunderland, MA.

[cit0046] Meuwissen T.H., Hayes B.J, and Goddard M.E 2001 Prediction of total genetic value using genome-wide dense marker maps. Genetics 157(4):1819–1829. 11290733 10.1093/genetics/157.4.1819 PMC1461589

[cit0047] Michaelson J.J., Alberts R, Schughart K, and Beyer A 2010 Data-driven assessment of eQTL mapping methods. BMC Genomics 11:502. doi:10.1186/1471-2164-11-502 20849587 10.1186/1471-2164-11-502 PMC2996998

[cit0048] Morota G., and Gianola D 2014 Kernel-based whole-genome prediction of complex traits: A review. Front. Genet. 5(10):1–13. 10.3389/fgene.2014.00363 PMC4199321 25360145

[cit0049] Motsinger-Reif A.A., Dudek S.M, Hahn L.W, and Ritchie M.D 2008 Comparison of approaches for machine-learning optimization of neural networks for detecting gene–gene interactions in genetic epidemiology. Genet. Epidemiol. 32(4):325–340. doi:10.1002/gepi.20307 10.1002/gepi.20307 18265411

[cit0050] Okechukwu R.U., and Dixon G.O 2008 Genetic gains from 30 years of cassava breeding in Nigeria for storage root yield and disease resistance in elite cassava genotypes. J. Crop Improv. 22(2):181–208. doi:10.1080/15427520802212506

[cit0051] Okogbenin E., Porto M, and Egesi C 2007 Marker-assisted introgression of resistance to cassava mosaic disease into Latin American germplasm for the genetic improvement of cassava in Africa. Crop Sci. 47:1895–1904. doi:10.2135/cropsci2006.10.0688

[cit0052] Oliveira E.J., Resende M.D.V, Silva Santos V, Ferreira C.F, Oliveira G.A.F, Silva M.S, et al 2012 Genome-wide selection in cassava. Euphytica 187(2):263–276. doi:10.1007/s10681-012-0722-0

[cit0053] Oliveira E.J., Santana F.A, Oliveira L.A, and Santos V.S 2014 Genetic parameters and prediction of genotypic values for root quality traits in cassava using REML/BLUP. Genet. Mol. Res. 13(3):6683–6700. doi:10.4238/2014.August.28.13 25177949 10.4238/2014.August.28.13

[cit0054] Olsen K.M., and Schaal B.A 1999 Evidence on the origin of cassava: Phylogeography of Manihot esculenta. Proc. Natl. Acad. Sci. USA 96(10):5586–5591. doi:10.1073/pnas.96.10.5586 10318928 10.1073/pnas.96.10.5586 PMC21904

[cit0055] Park T., and Casella G 2008 The Bayesian Lasso. J. Am. Stat. Assoc. 103(482):681–686. doi:10.1198/016214508000000337

[cit0056] Parkes E., Fregene M, Dixon A, Okogbenin E, Boakye-Peprah B, and Labuschagne M.T. 2015 Developing Cassava Mosaic Disease resistant cassava varieties in Ghana using a marker assisted selection approach. Euphytica 203:549–556. doi:10.1007/s10681-014-1262-6

[cit0057] Perez-Rodriguez P., Gianola D, Gonzalez-Camacho J.M, Crossa J, Manes Y, and Dreisigacker S 2013 Comparison between linear and non-parametric regression models for genome-enabled prediction in wheat. G3 (Bethesda) 2(12):1595–1605. 10.1534/g3.112.003665 PMC3516481 23275882

[cit0058] Pérez P., and De Los Campos G 2014 Genome-wide regression and prediction with the BGLR statistical package. Genetics 198(2):483–495. doi:10.1534/genetics.114.164442 25009151 10.1534/genetics.114.164442 PMC4196607

[cit0059] Plummer M., Best N, Cowles K, and Vines K 2006 CODA: Convergence diagnosis and output analysis for MCMC. R News 6(March):7–11.

[cit0060] Rabbi I.Y., Hamblin M.T, Kumar P.L, Gedil M.A, Ikpan A.S, Jannink J.-L, et al 2014 High-resolution mapping of resistance to cassava mosaic geminiviruses in cassava using genotyping-by-sequencing and its implications for breeding. Virus Res. 186:87–96. doi:10.1016/j.virusres.2013.12.028 24389096 10.1016/j.virusres.2013.12.028

[cit0061] Resende M.F.R., Munoz P, Resende M.D.V, Garrick D.J, Fernando R.L, Davis J.M, et al 2012 Accuracy of genomic selection methods in a standard data set of loblolly pine (Pinus taeda L.). Genetics 190(4):1503–1510. doi:10.1534/genetics.111.137026 22271763 10.1534/genetics.111.137026 PMC3316659

[cit0062] Rincent R., Laloë D, Nicolas S, Altmann T, Brunel D, Revilla P, et al 2012 Maximizing the reliability of genomic selection by optimizing the calibration set of reference individuals: Comparison of methods in two diverse groups of maize inbreds (Zea mays L.). Genetics 192(2):715–728. doi:10.1534/genetics.112.141473 22865733 10.1534/genetics.112.141473 PMC3454892

[cit0063] Rutkoski J.E., Poland J.A, Singh R.P, Huerta-Espino J, Bhavani S, Barbier H, et al 2014 Genomic selection for quantitative adult plant stem rust resistance in wheat. Plant Genome 7(3) doi:10.3835/plantgenome2014.02.0006

[cit0064] Rutkoski J., Singh R, and Huerta-Espino J 2015 Efficient use of historical data for genomic selection: A case study of stem rust resistance in wheat. Plant Genome 8(1). doi:10.3835/plantgenome2014.09.0046 10.3835/plantgenome2014.09.0046 33228293

[cit0065] Sarkar R.K., Rao A.R, Meher P.K, Nepolean T, and Mohapatra T 2015 Evaluation of random forest regression for prediction of breeding value from genomewide SNPs. J. Genet. 94(2):187–192. doi:10.1007/s12041-015-0501-5 26174666 10.1007/s12041-015-0501-5

[cit0066] Spindel J., Begum H, Akdemir D, Virk P, Collard B, Redona E, et al 2015 Genomic selection and association mapping in rice (Oryza sativa): Effect of trait genetic architecture, training population composition, marker number and statistical model on accuracy of rice genomic selection in elite, tropical rice breeding lines. PLoS Genet. 11(2):E1004982. doi:10.1371/journal.pgen.1004982 25689273 10.1371/journal.pgen.1004982 PMC4334555

[cit0067] Strobl C., Malley J, and Tutz G 2009 An Introduction to recursive partitioning: Rationale, application and characteristics of classification and regression trees, bagging and random forests. Psychol. Methods 14(4):323–348. doi:10.1037/a0016973 19968396 10.1037/a0016973 PMC2927982

[cit0068] Su G., Christensen O.F, Janss L, and Lund M.S 2014 Comparison of genomic predictions using genomic relationship matrices built with different weighting factors to account for locus-specific variances. J. Dairy Sci. 97(10):6547–6559. doi:10.3168/jds.2014-8210 25129495 10.3168/jds.2014-8210

[cit0069] Sungthongw K., Promkhambu A, Laoken A, and Polthanee A 2016 Effects of methods and duration storage on cassava stake characteristics. Asian J. Plant Sci. 15(3):86–91. doi:10.3923/ajps.2016.86.91

[cit0070] VanRaden P.M 2008 Efficient methods to compute genomic predictions. J. Dairy Sci. 91(11):4414–4423. doi:10.3168/jds.2007-0980 18946147 10.3168/jds.2007-0980

[cit0071] Wolfe M.D., Kulakow P, Rabbi I.Y, and Jannink J.-L 2016a Markerbased estimates reveal Significant non-additive effects in clonally propagated cassava (Manihot esculenta): Implications for the prediction of total genetic value and the selection of varieties. G3 (Bethesda) 6(11):3497–3506. doi:10.1534/g3.116.033332. 27587297 10.1534/g3.116.033332 PMC5100848

[cit0072] Wolfe M.D., Rabbi I.Y, Egesi C, Hamblin M, Kawuki R, Kulakow P, et al 2016b Genome-wide association and prediction reveals the genetic architecture of cassava mosaic disease resistance and prospects for rapid genetic improvement. Plant Genome 9(2):1–13. doi:10.3835/plantgenome2015.11.0118 10.3835/plantgenome2015.11.0118 27898832

