# Supplementary material for: Prospects for Genomic Selection in Cassava Breeding
Source: Plant Genome. 2017 Sep 28;10(3):10.3835/plantgenome2017.03.0015. doi: 10.3835/plantgenome2017.03.0015 (PMC7822052; doi:10.3835/plantgenome2017.03.0015)
Supplement: Supplementary file 1 [file TPG-10-03-0015-s001.pdf]

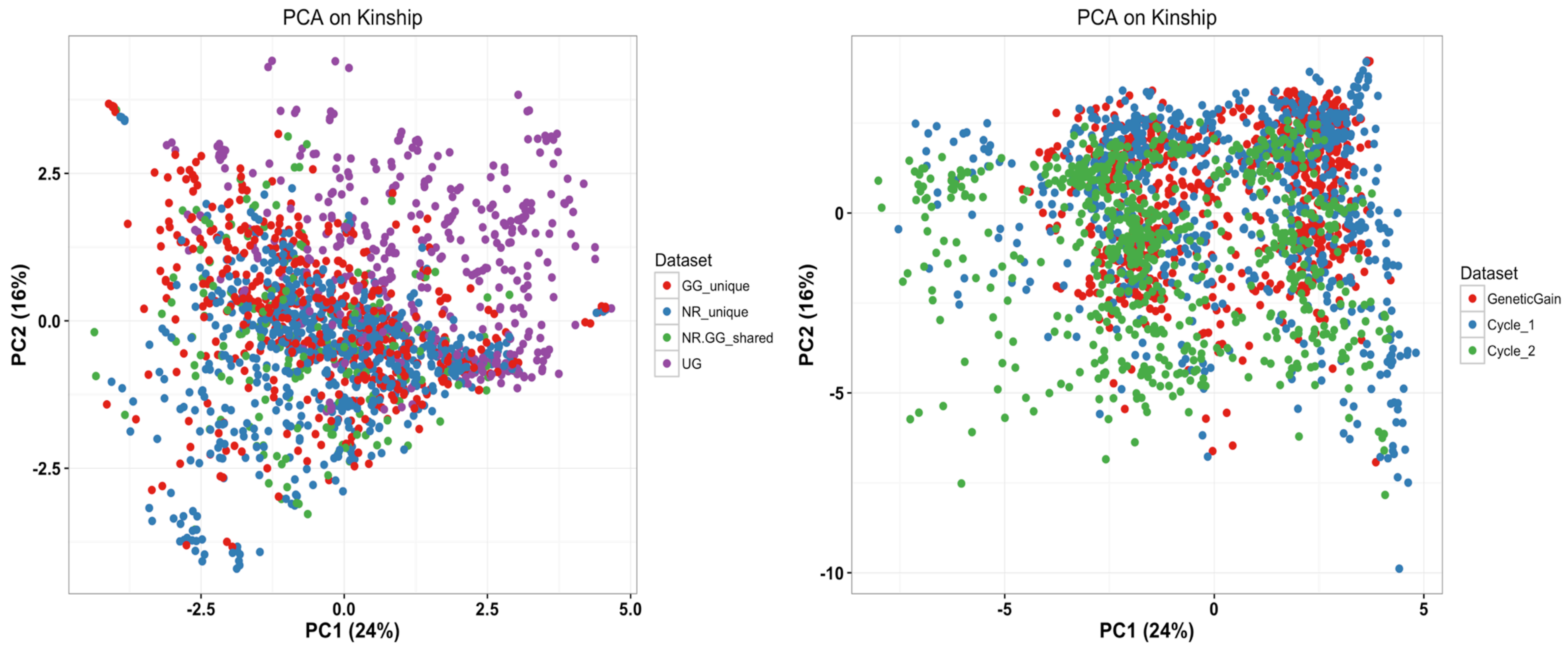

A. Three Training Populations **B.** IITA Genomic Selection Program

Supplementary Figure 1. Population genetic structure of the datasets analyzed in this study, illustrated by plotting the first two components (PCs), following principal components analysis (PCA) of the genetic relationship matrix. The training populations for the each breeding institutes genomic selection program are compared on the left **(A)**. The breeding cycles (Genetic Gain, Cycle 1 and Cycle 2) from the IITA genomic selection program are contrasted on the right **(B)**.

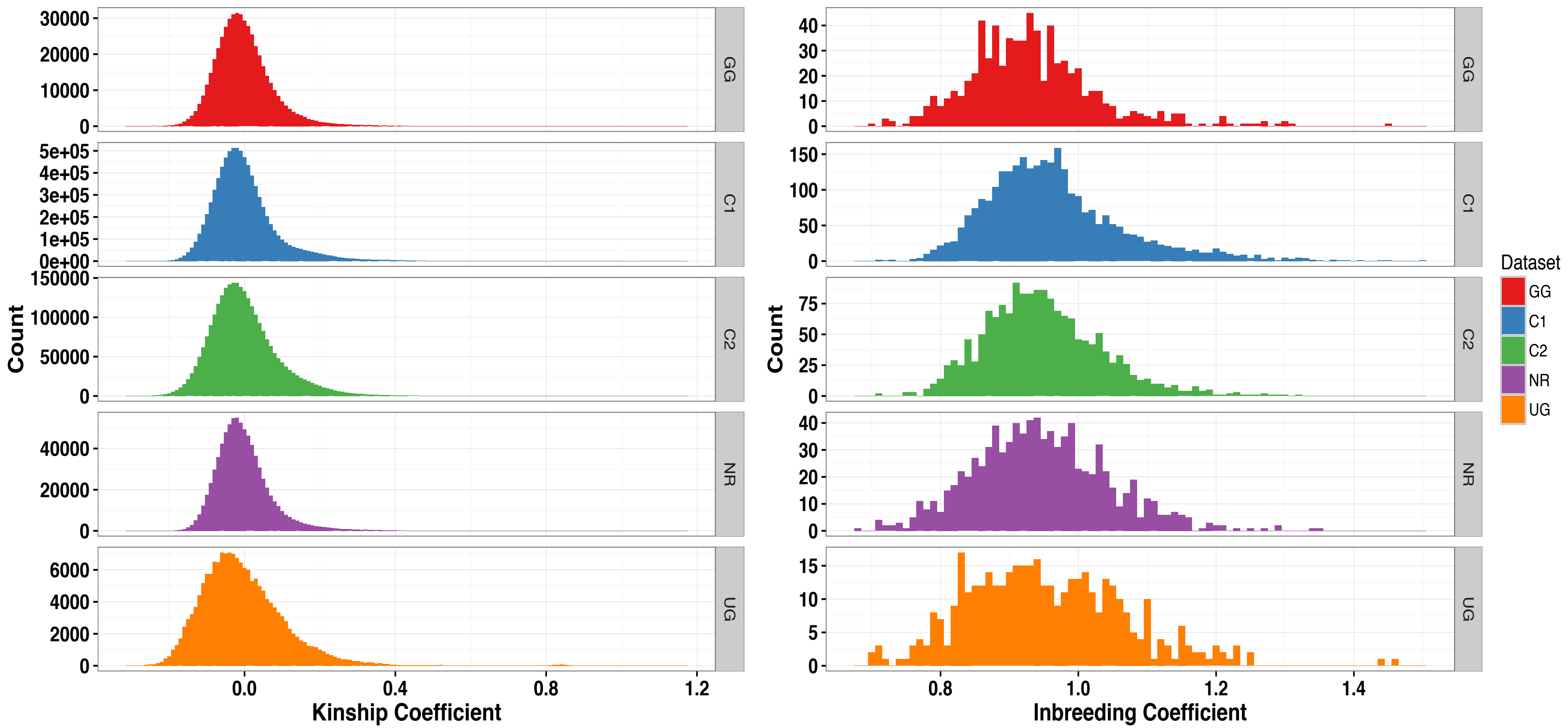

Supplementary Figure 2. Histogram of kinship coefficients (off-diagonals of genomic relationship matrix [GRM]) on left and inbreeding values (diagonals of GRM) on the right for all five datasets analyzed in this study. GRM constructed for each dataset separately with markers with >1% minor allele frequency. IITA = International Institute of Tropical Agriculture; GG = IITA Genetic Gain; C1 = IITA Cycle 1; C2 = IITA Cycle 2; NR = National Root Crops Research Institute; UG = National Crops Research Resources Institute.

Supplementary Figure 3. GS Cross-validation accuracies in the NaCRRI dataset. Five fold cross validation results for seven traits using GBLUP, RKHS (GAUSS_K0.01 = single RKHS kernel, MultiKernelGauss =Multi-kernel RKHS), BayesA, BayesB, BayesC, BL = Bayesian Lasso and Random Forest. DM = dry matter content; HI = harvest index; RTWT = root weight; RTNO = root number; SHTWT = shoot weight; MCMDS = mean cassava mosaic disease severity; VIGOR = early plant vigor.

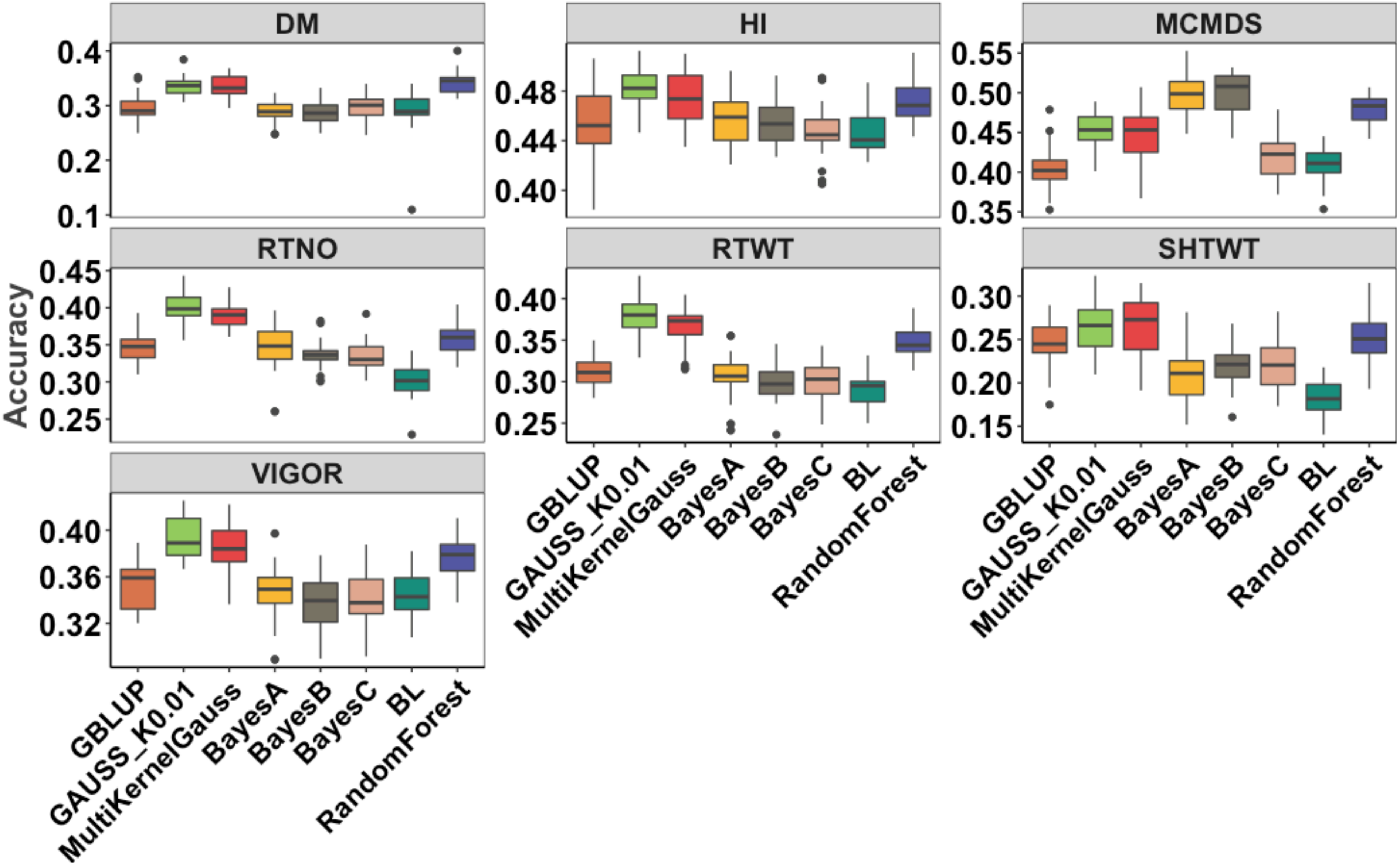

Supplementary Figure 4. GS Cross-validation accuracies in the NRCRI dataset. Five fold cross validation results for seven traits using GBLUP, RKHS (GAUSS_K0.01 = single RKHS kernel, MultiKernelGauss =Multi-kernel RKHS), BayesA, BayesB, BayesC, BL = Bayesian Lasso and Random Forest. DM = dry matter content; HI = harvest index; RTWT = root weight; RTNO = root number; SHTWT = shoot weight; MCMDS = mean cassava mosaic disease severity; VIGOR = early plant vigor.

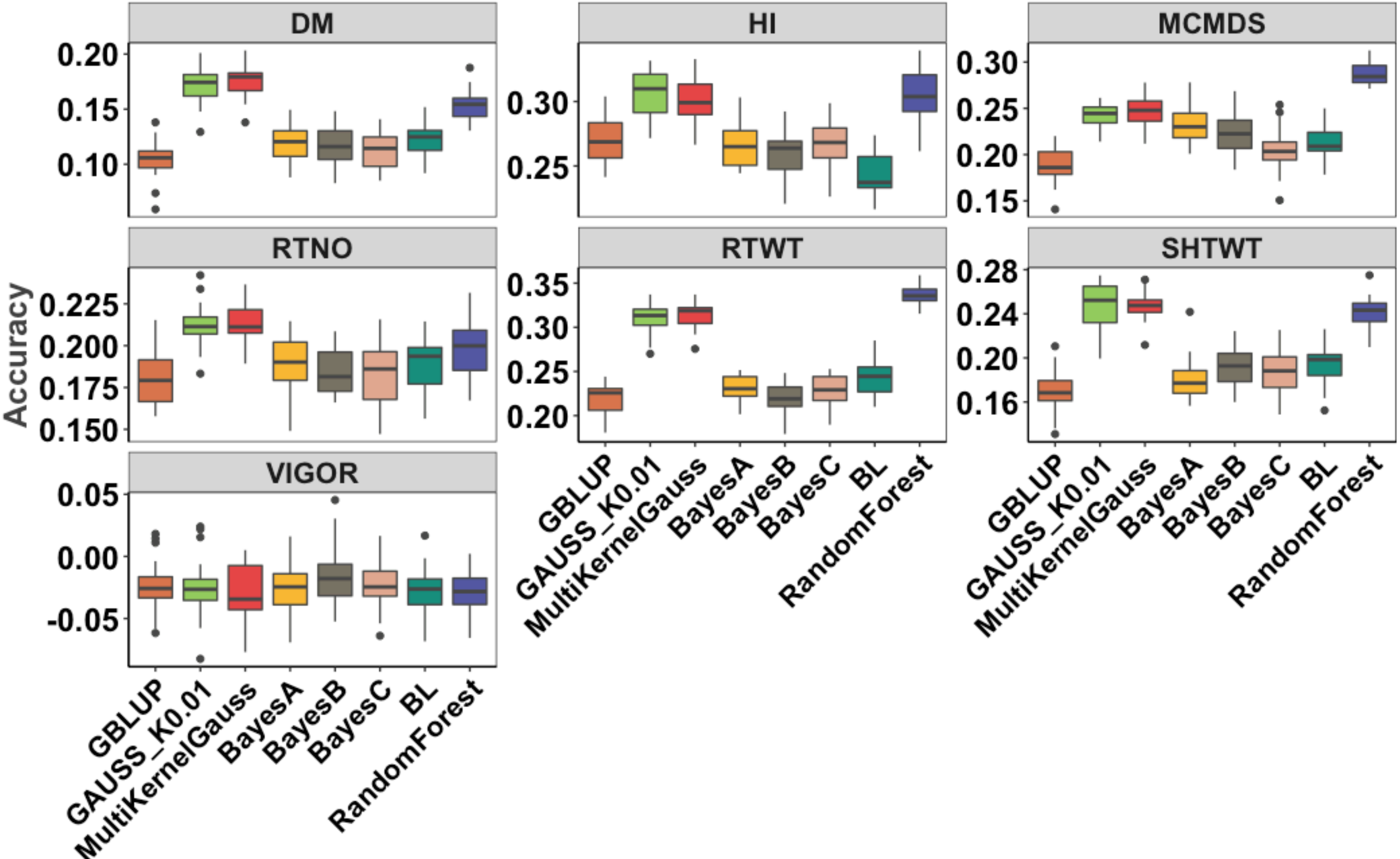

Supplementary Figure 5. GS Cross-validation accuracies in the Genetic Gain dataset. Five fold cross validation results for seven traits using GBLUP, RKHS (GAUSS_K0.01 = single RKHS kernel, MultiKernelGauss =Multi-kernel RKHS), BayesA, BayesB, BayesC, BL = Bayesian Lasso and Random Forest. DM = dry matter content; HI = harvest index; RTWT = root weight; RTNO = root number; SHTWT = shoot weight; MCMDS = mean cassava mosaic disease severity; VIGOR = early plant vigor.

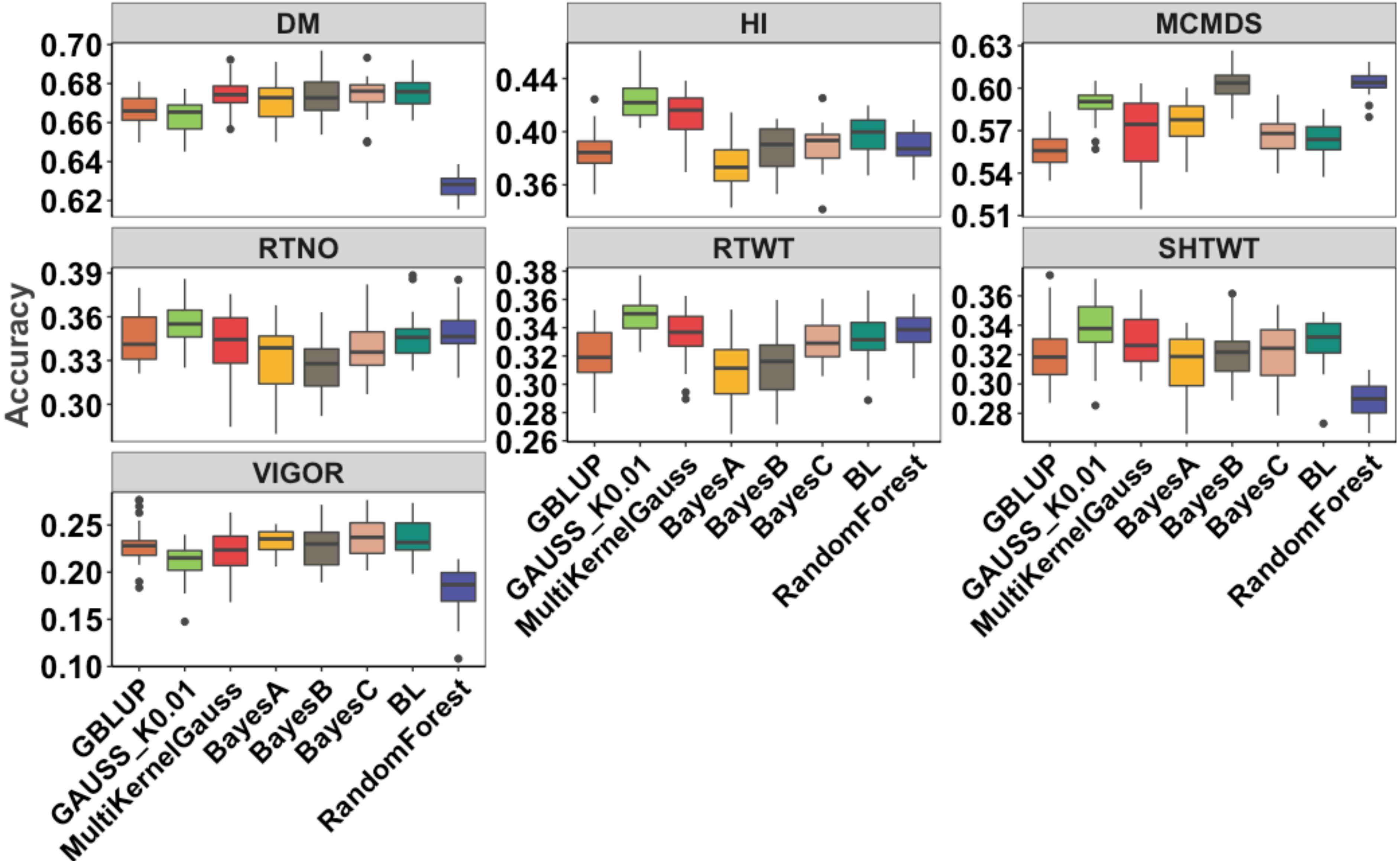

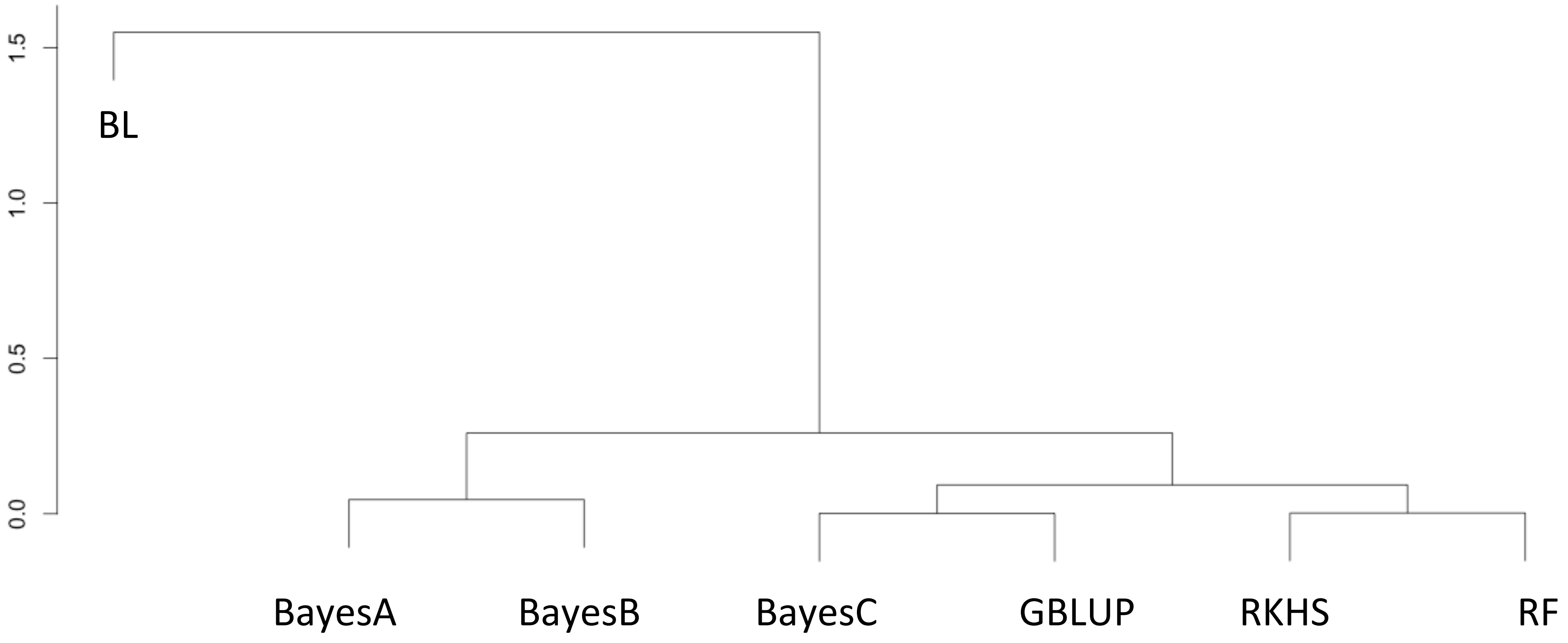

Supplementary Figure 6. Hierarchical clustering of genomic prediction models based on cross-validated genomic estimated breeding values (GEBVs). Height on the y-axis refers to the value of the dissimilarity criterion. Clustering of prediction models in combined results for all populations. GBLUP= genomic best linear unbiased predictor; BL = Bayesian Lasso; RF = random forest; RKHS = reproducing kernel Hilbert space (multi-kernel model).

Across population optimization NaCRRI

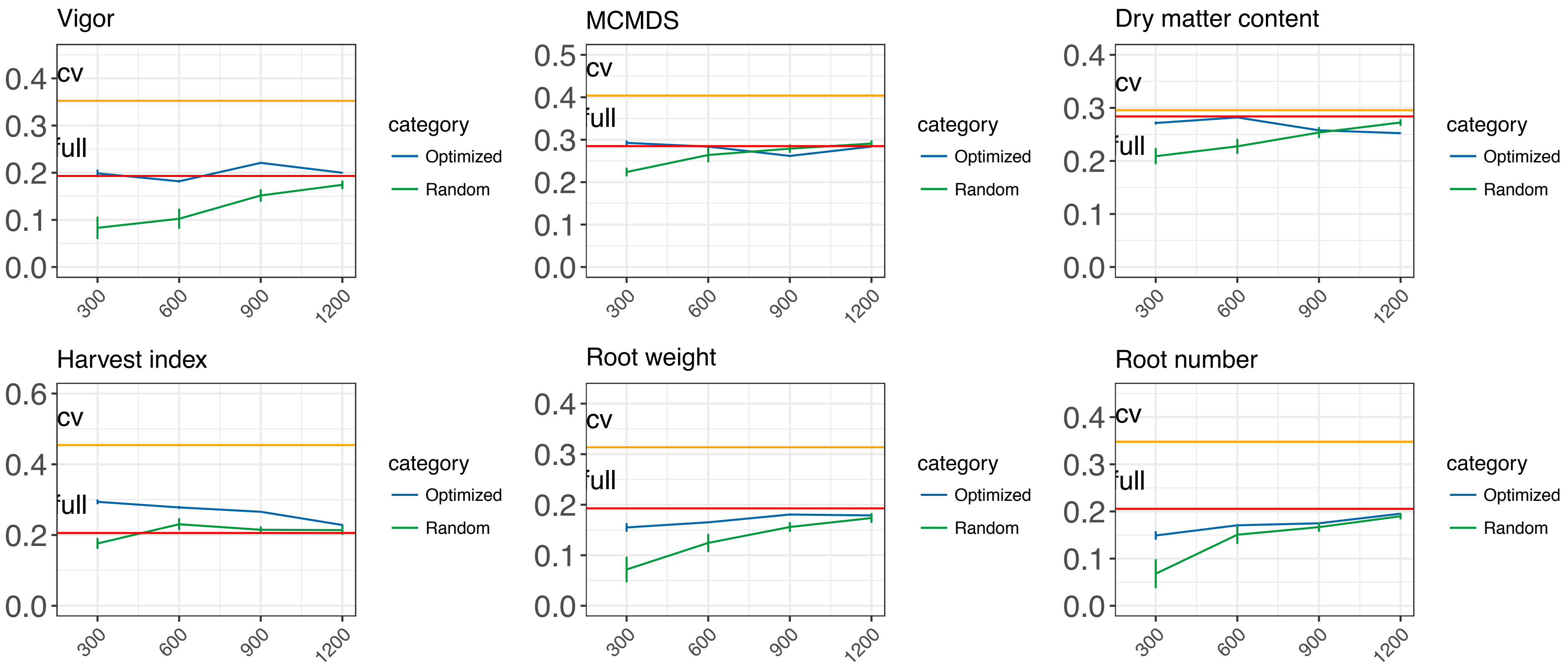

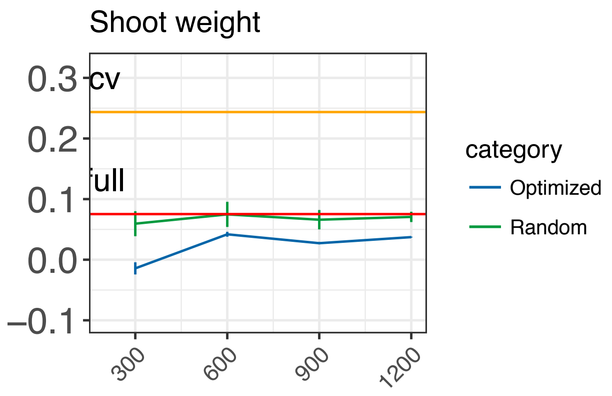

Supplementary Figure 7. Cross population prediction of UG. The accuracy of prediction for seven traits with the combined NR+GG population training data. Subsets sizes(x-axis) were selected either at random or using the genetic algorithm implemented in the R package STPGA. Ten random and ten STPGA-selected subsets were made at each training set size. Error bars are the standard error around the mean for the ten samples. Horizontal lines show the mean cross-validation accuracy for the UG population (validation set; orange line) and the accuracy of the full set of NR+GG predicting the UG population (red line).

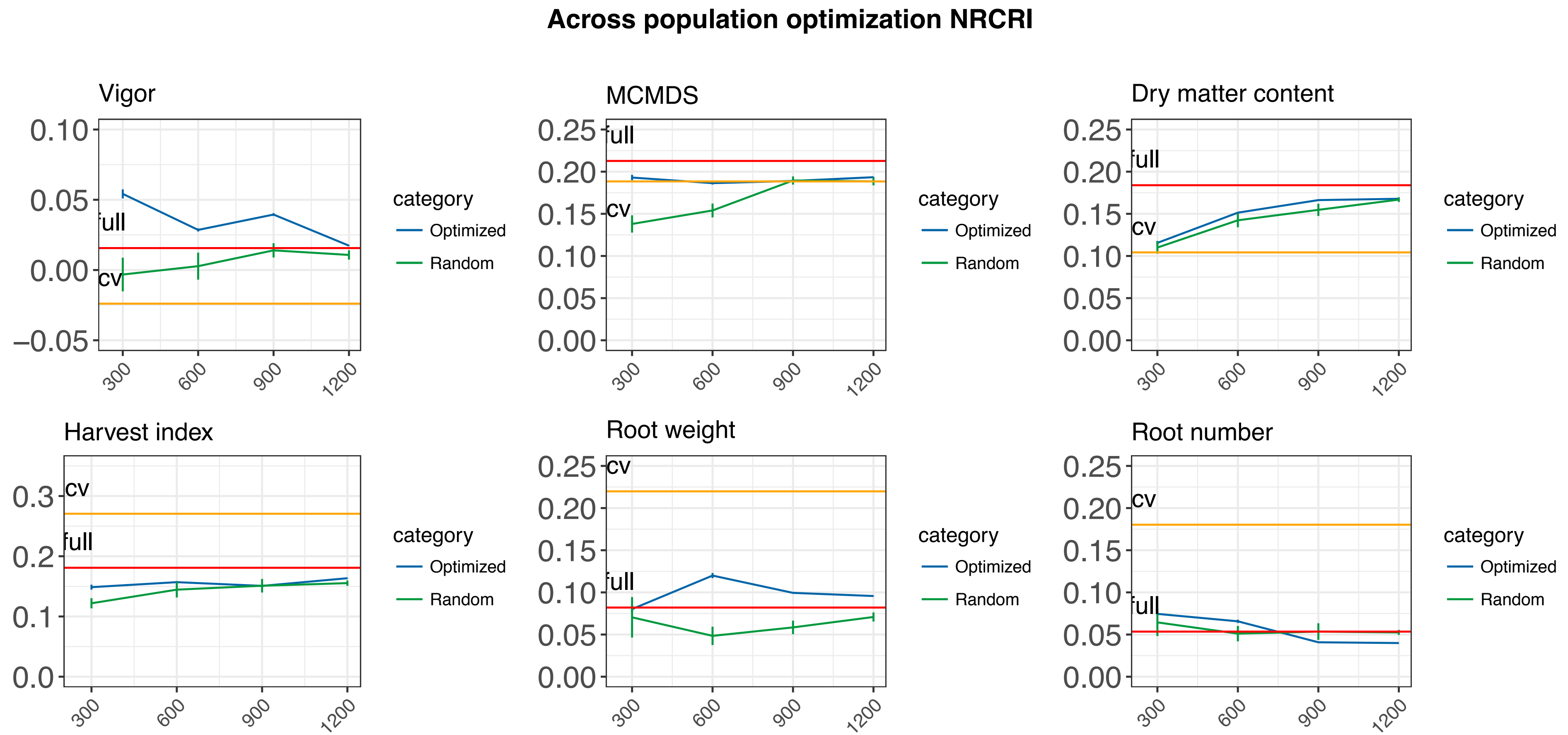

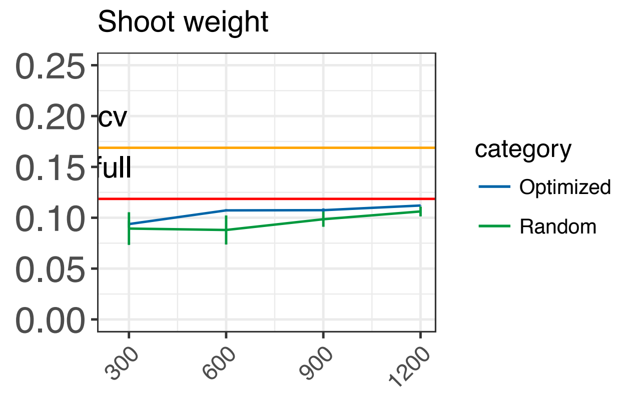

Supplementary Figure 8. Cross population prediction of NR. The accuracy of prediction for seven traits with the combined UG+GG population training data. Subsets sizes(x-axis) were selected either at random or using the genetic algorithm implemented in the R package STPGA. Ten random and ten STPGA-selected subsets were made at each training set size. Error bars are the standard error around the mean for the ten samples. Horizontal lines show the mean cross-validation accuracy for the NRCRI population (validation set; orange line) and the accuracy of the full set of UG +GG predicting the NR population (red line).

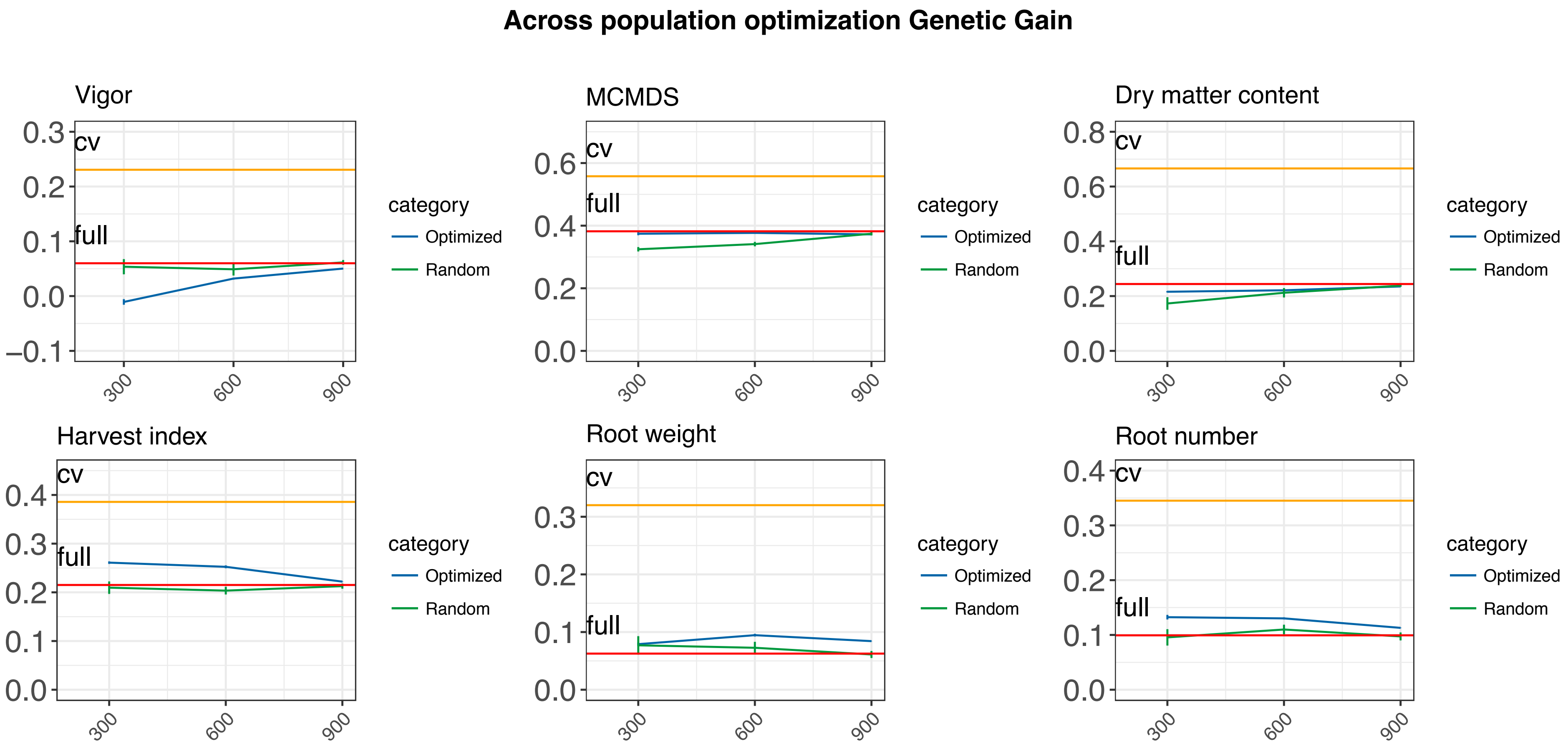

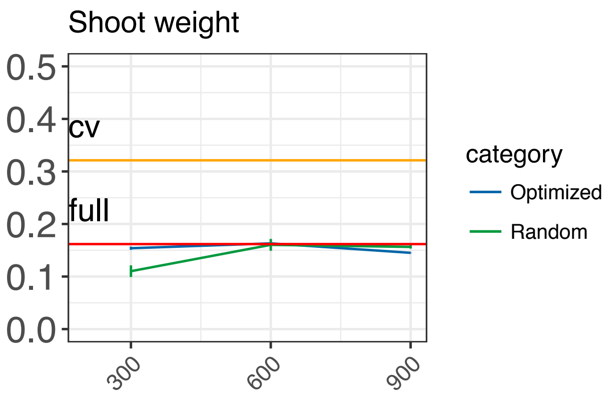

Supplementary Figure 9. Cross population prediction of GG. The accuracy of prediction for seven traits with the combined NR+UG population training data. Subsets sizes(x-axis) were selected either at random or using the genetic algorithm implemented in the R package STPGA. Ten random and ten STPGA-selected subsets were made at each training set size. Error bars are the standard error around the mean for the ten samples. Horizontal lines show the mean cross-validation accuracy for the GG population (validation set; orange line) and the accuracy of the full set of NR+UG predicting the GG population (red line).

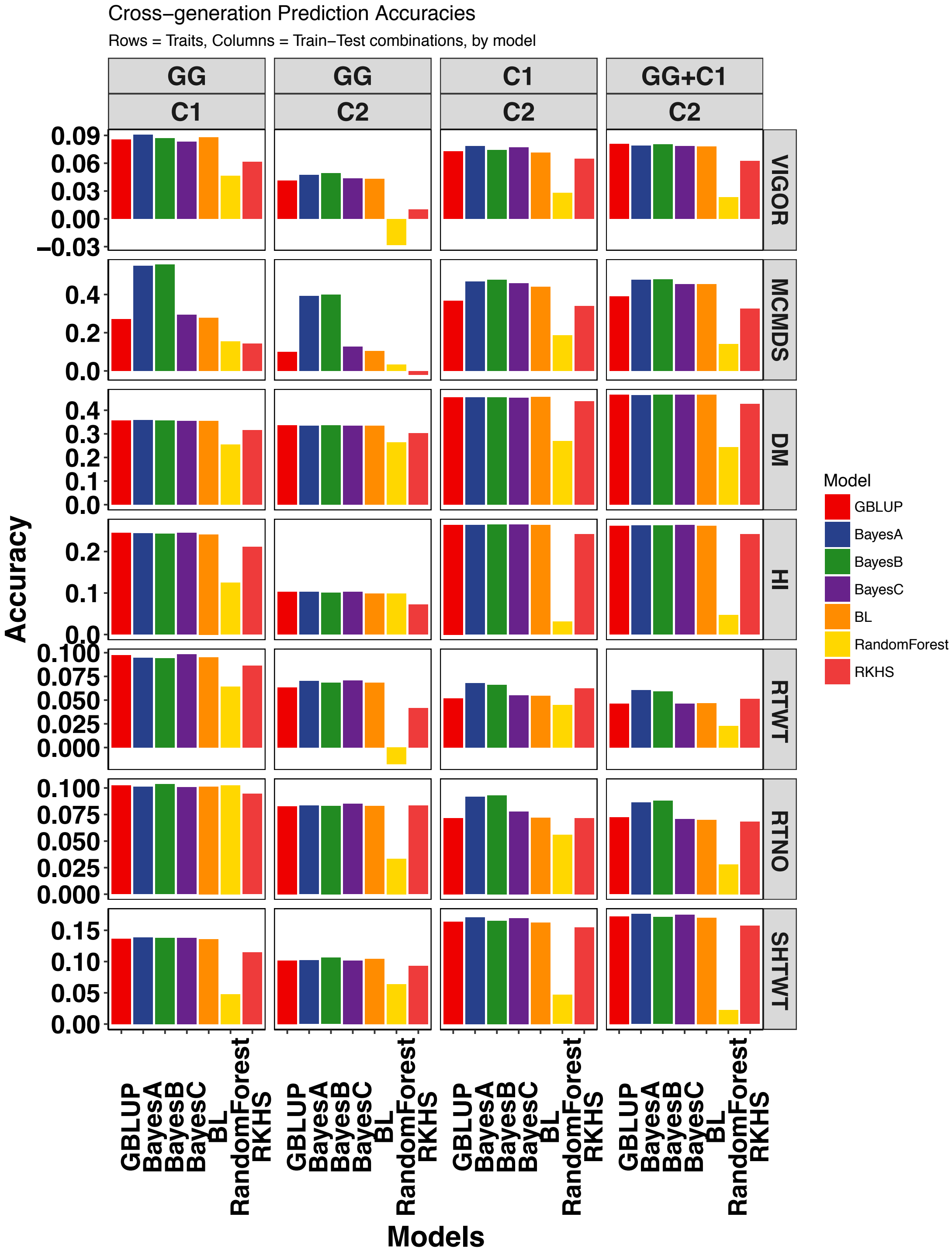

Supplementary Figure 10. Cross-generation prediction accuracies. In the IITA genomic selection dataset there are three generations of clones: the Genetic Gain (GG), their progeny the Cycle 1 (C1) and C1's progeny, the Cycle 2 (C2). For each of seven traits (rows) and seven prediction models (x-axis, colors) we made four across-generation predictions (columns): GG predicts C1, GG predicts C2, C1 predicts C2, GG +C1 predicts C2. DM = dry matter content; HI = harvest index; RTWT = root weight; RTNO = root number; SHTWT = shoot weight; MCMDS = mean cassava mosaic disease severity; VIGOR = early plant vigor.

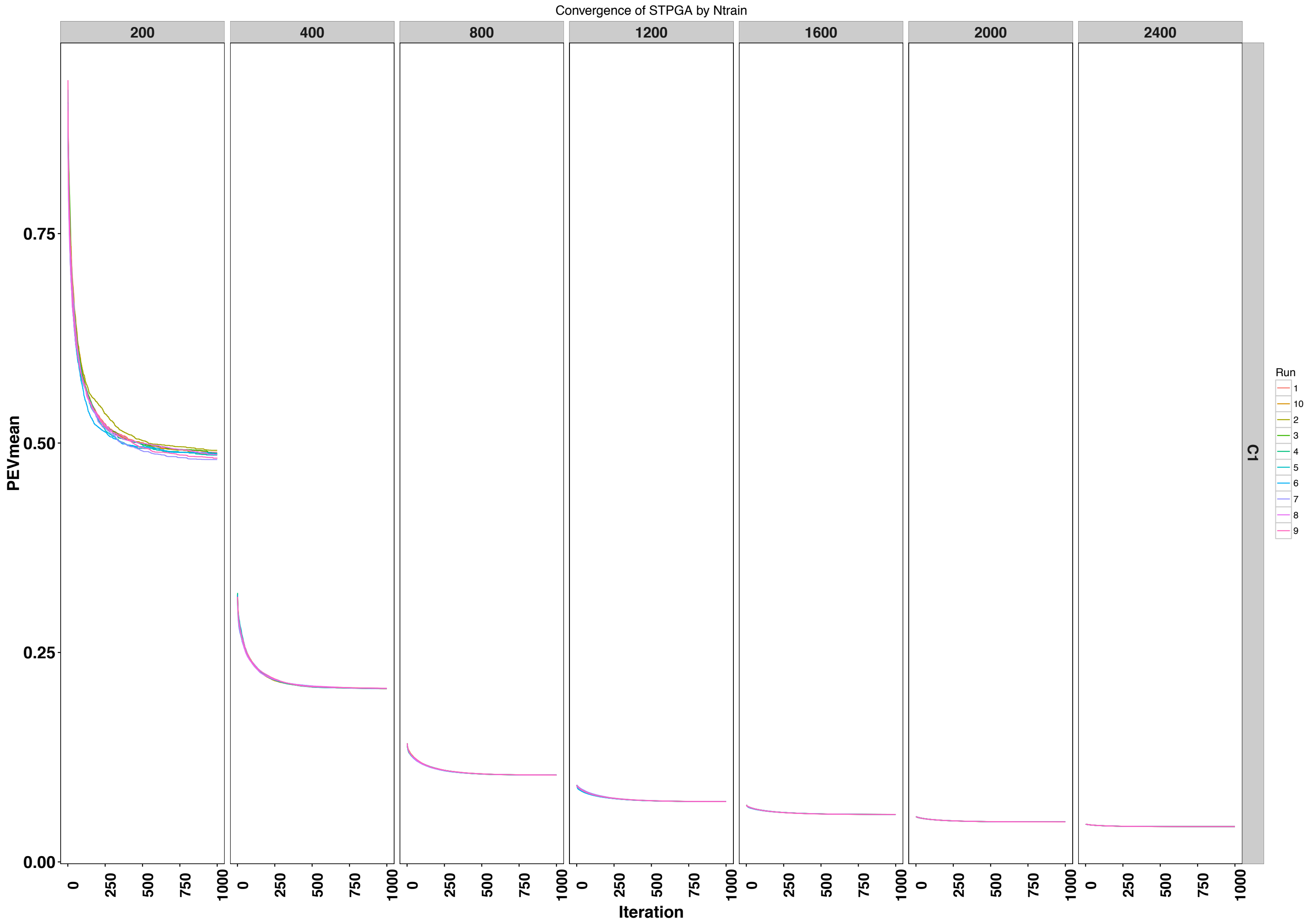

Supplementary Figure 11. Convergence of the genetic algorithm implemented in the R package STPGA. Plot of the optimization criterion (PEVmean, y-axis) vs. the iteration of the genetic algorithm (x-axis) across training sample sizes (panels). Samples were drawn from the IITA Cycle 1 (C1), excluding the parents of cycle 2 (PofC2). The algorithm was set to find the smallest PEVmean with the PofC2 as the test (validation) set and a sample of the C1 as the training set. Ten runs of the genetic algorithm are shown in different colored lines.

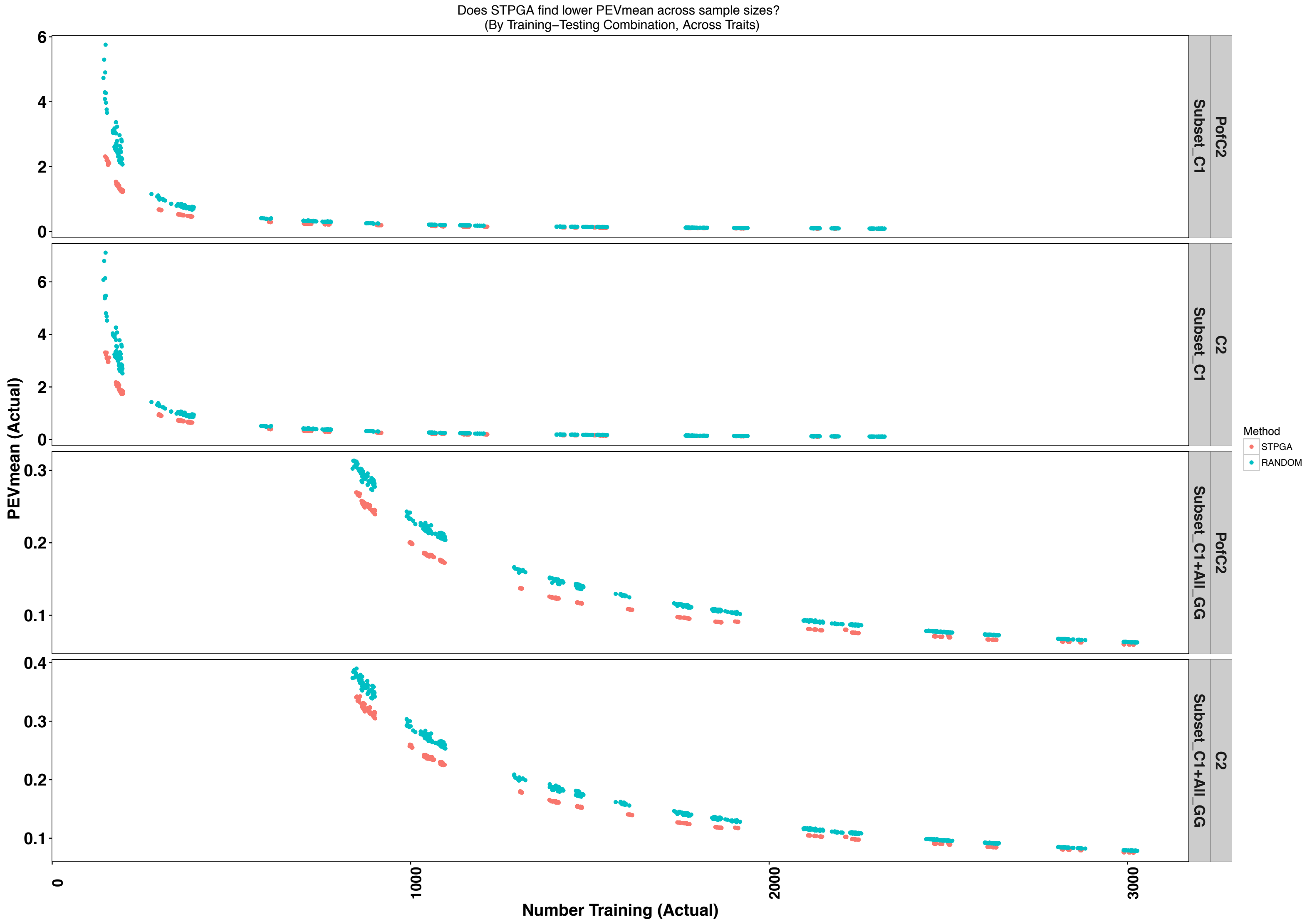

Supplementary Figure 12. Does STPGA find lower PEVmean across sample sizes compared to random? The size of training samples used in four prediction scenarios (rows) is plotted against the PEVmean of the subset for every trait and each of 10 samples elected either by the genetic algorithm implemented in the R package STPGA (red) or randomly (blue). The actual PEVmean and number of training samples are plotted here, with variations from planned sample sizes and PEVmeans initially expected due to missing data for some traits or individuals.

The genetic algorithm implemented by STPGA was run ten times. The validation set target for the optimization algorithm were the parents of IITA's Cycle 2 (PofC2) and the training sets were samples of differing size of the IITA Cycle 1 (C1). Predictions were made either with samples of C1 only (rows 1 and 2) or with samples of C1 plus the entire GG (rows 3 and 4). Validation sets were either the PofC2 (rows 1 and 3) or the C2 (rows 2 and 4).

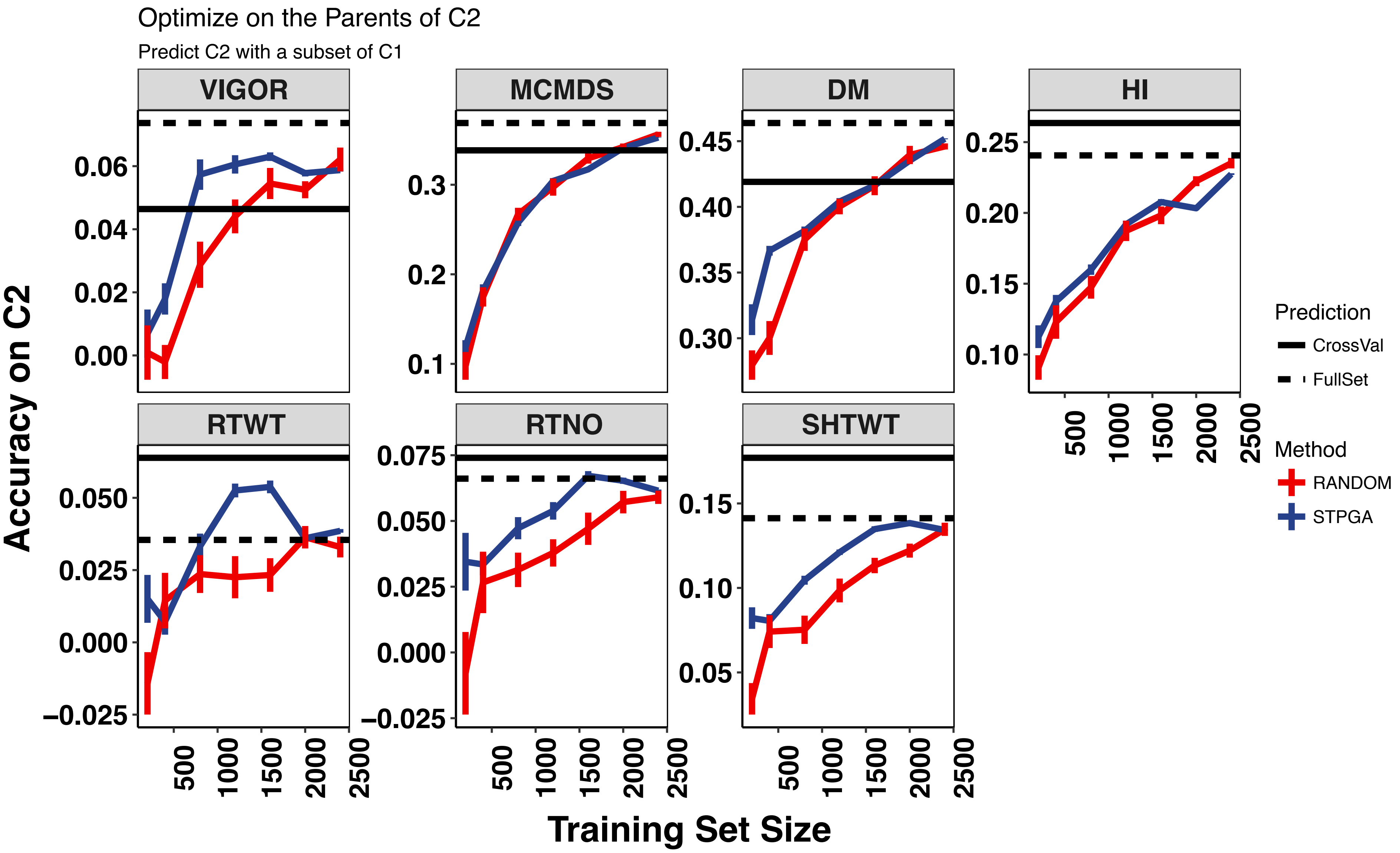

Supplementary Figure 13. The relationship between training set size and accuracy predicting IITA Cycle 2 (across-generation). The accuracy of prediction for seven traits (panels) with different size subsets (x-axis) of the IITA Cycle 1 (C1) is shown. Subsets of a given size were selected either at random or using the genetic algorithm implemented in the R package STPGA. Ten random and ten STPGA-selected subsets were made at each training set size. Error bars are the standard error around the mean for the ten samples. Horizontal black lines show the mean cross-validation accuracy for the C2 (validation set; solid line) and the accuracy of the full set of GG+C1 predicting C2 (dashed line). GBLUP was used for all predictions. DM = dry matter content; HI = harvest index; RTWT = root weight; RTNO = root number; SHTWT = shoot weight; MCMDS = mean cassava mosaic disease severity; VIGOR = early plant vigor.

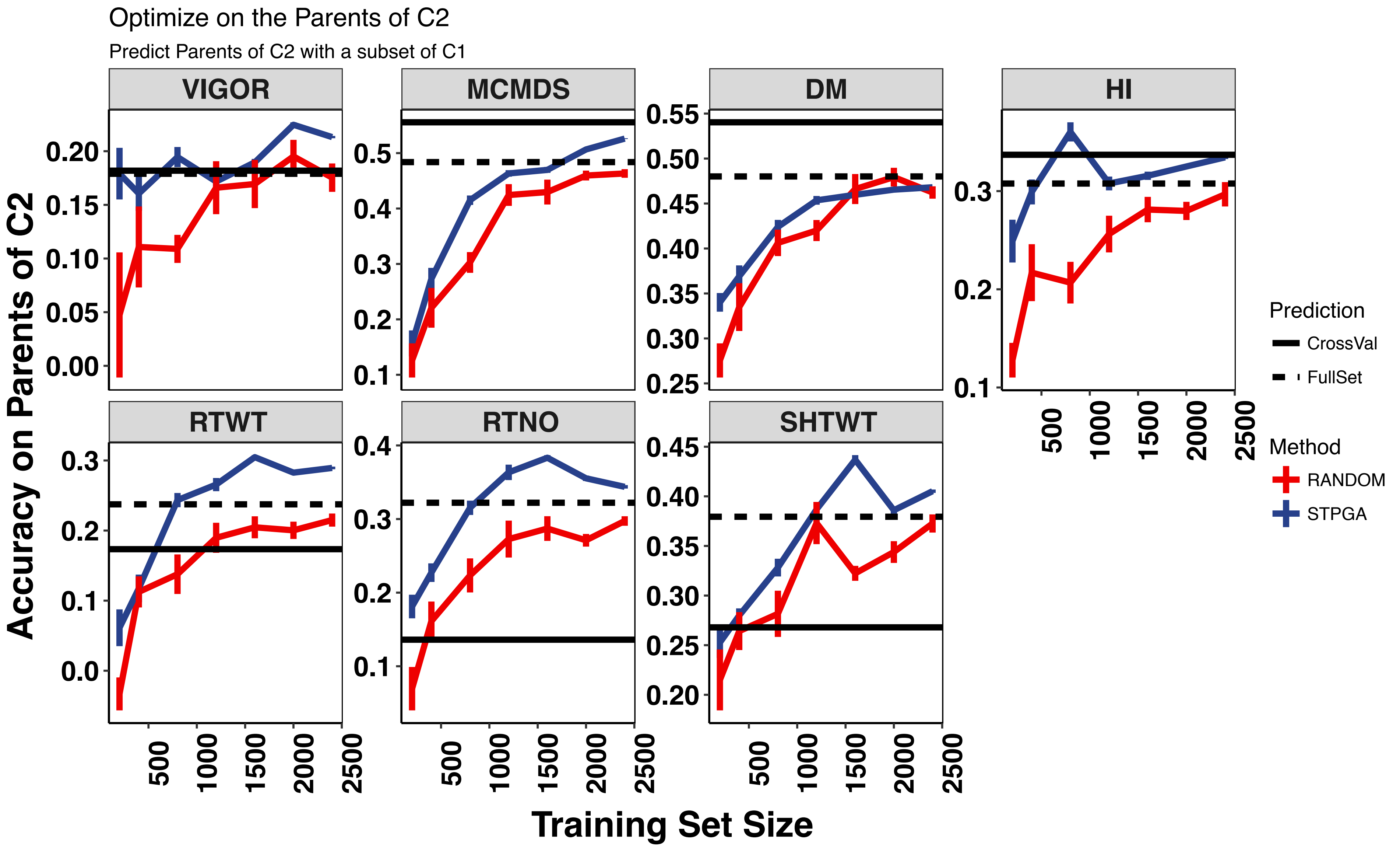

Supplementary Figure 14. The relationship between training set size and accuracy predicting the parents of Cycle 2 (from Cycle 1, within-generation). The accuracy of prediction for seven traits (panels) with different size subsets (x-axis) of the IITA Cycle 1 (C1) is shown. Subsets of a given size were selected either at random or using the genetic algorithm implemented in the R package STPGA. Ten random and ten STPGA-selected subsets were made at each training set size. Error bars are the standard error around the mean for the ten samples. Horizontal black lines show the mean cross-validation accuracy for the C1 (validation set; solid line) and the accuracy of the full set of GG+C1 predicting the parents of C2 (dashed line). GBLUP was used for all predictions. DM = dry matter content; HI = harvest index; RTWT = root weight; RTNO = root number; SHTWT = shoot weight; MCMDS = mean cassava mosaic disease severity; VIGOR = early plant vigor.
