# Supplementary material for: Prospects for Genomic Selection in Cassava Breeding
Source: Plant Genome. 2017 Sep 28;10(3):10.3835/plantgenome2017.03.0015. doi: 10.3835/plantgenome2017.03.0015 (PMC7822052; doi:10.3835/plantgenome2017.03.0015)
Supplement: Supplementary file 2 [file TPG-10-03-0015-s002.pdf]

SUPPLEMENTARY METHODS

Phenotyping Trial Design

Clones from the NaCRRI collection were trialed in two seasons, 2012-2013 and 2013-2014 in Uganda, yielding a total 2391 observations with plots of 10 plants each. In the first year, 288 clones were planted in a single location without replication or augmentation but with 14 incomplete blocks. In the following field season, the number of clones expanded to 411, planted in 3 locations (Namulonge, Kasese and Ngetta) with two replications each. In addition, field variability is accounted for in this year by a variable "RANGE", which is equivalent to row or column and is further sub-divided into blocks.

The NRCRI clones were phenotyped in 2012-2013 and 2013-2014 field seasons. In 2012-2013 an initial trial, called "TP1" of 502 clones was established in Umudike, Nigeria. The trial was replicated three times and replications were further subdivided into incomplete blocks. In 2013, TP1 was planted again, with 519 clones (23 new genotypes) and in three locations (Umudike, Kano and Otobi). This time, the trial was planted in two phases, which have been labeled "sets" with approximately ½ of the clones in each, making the "set" variable a form of blocking factor within each location. Each "set" then contained three replicates and replications were again subdivided into incomplete blocks. In addition, a second trial, called "TP2" was planted in 2013 containing 486 clones (49 shared with TP1. The TP2 trial was planted only in Umudike, in four sets, with three replications each, subdivided into incomplete blocks like TP1. In total there were 7662 observations from NRCRI trials with plots of five stands each.

The IITA dataset contains 3 sets of trials, all of which were conducted in the 2013-2014 and 2014-2015 field seasons in Nigeria. The Genetic Gain (GG) trials (N=2121) are an augmented design with two checks (clones TMEB1 and I30572) and plots of 10 plants each. The trials were conducted in Ubiaja and Ibadan in 2013-2014 and Ubiaja only in 2014-2015. The second set of trials is also augmented and contains genomic selection progenies as well as some GG clones.

In 2013-2014, 2176 C1 clones derived from the 2013 seedling nursery and 112 GG clones were phenotyped in three locations (Ibadan, Ikenne and Mokwa) with five plants per plot. There were not enough materials to replicate clones across locations. Instead, families were replicated in an approximately balanced fashion in each location.

In 2014-2015, a subset of 805 of the C1 clones trialed in the previous year and 37 GG clones were phenotyped again in the same three locations with the same experimental design except the plot size was increased to 20 plants each. In the same year, another trial was planted in the same locations and with the same basic design but with 5 plants per clone. This trial was planted containing 1944 clones, 1441 from C2, 524 C1 from the 2014 C1 seedling nursery and 9 GG.

Finally, we have included data from crossing blocks collected in 2013 and 2014. All crossing blocks are planted in Ubiaja, Nigeria. Data from these trials are the only source of phenotypes for C1 and C2 clones that were selected to be parents. One crossing block was planted in 2013 with 226 clones, 68 from GG and 158 from the 2013-derived C1 with 10 plant plots. Two crossing blocks were planted in 2014, one had 20 plant plots with 219 clones (151 C1 from the 2013 seedling nursery and 68 GG) and the other had 173 clones (46 C1 from the 2014 seedling nursery and 127 C2) in 10 plant plots.
